# Investigating non-inferiority or equivalence in time-to-event data under non-proportional hazards

**DOI:** 10.1007/s10985-023-09589-5

**Published:** 2023-01-28

**Authors:** Kathrin Möllenhoff, Achim Tresch

**Affiliations:** 1 Mathematical Institute, Heinrich Heine University, 40225 Düsseldorf, Germany; 2 Institute of Medical Statistics and Computational Biology, Faculty of Medicine, University of Cologne, Cologne, Germany; 3 CEDAD, University of Cologne, Cologne, Germany; 4 Center for Data and Simulation Science, University of Cologne, Cologne, Germany

**Keywords:** Equivalence, Non-inferiority, Non-proportional hazards, Survival analysis, Time-to-event data

## Abstract

The classical approach to analyze time-to-event data, e.g. in clinical trials, is to fit Kaplan–Meier curves yielding the treatment effect as the hazard ratio between treatment groups. Afterwards, a log-rank test is commonly performed to investigate whether there is a difference in survival or, depending on additional covariates, a Cox proportional hazard model is used. However, in numerous trials these approaches fail due to the presence of non-proportional hazards, resulting in difficulties of interpreting the hazard ratio and a loss of power. When considering equivalence or non-inferiority trials, the commonly performed log-rank based tests are similarly affected by a violation of this assumption. Here we propose a parametric framework to assess equivalence or non-inferiority for survival data. We derive pointwise confidence bands for both, the hazard ratio and the difference of the survival curves. Further we propose a test procedure addressing non-inferiority and equivalence by directly comparing the survival functions at certain time points or over an entire range of time. Once the model’s suitability is proven the method provides a noticeable power benefit, irrespectively of the shape of the hazard ratio. On the other hand, model selection should be carried out carefully as misspecification may cause type I error inflation in some situations. We investigate the robustness and demonstrate the advantages and disadvantages of the proposed methods by means of a simulation study. Finally, we demonstrate the validity of the methods by a clinical trial example.

**Supplementary Information:**

The online version contains supplementary material available at 10.1007/s10985-023-09589-5.

## Introduction

Time-to-event outcomes are frequently observed in medical research, for instance in the area of oncology or cardiovascular diseases. A commonly addressed issue is the comparison of a test to a reference treatment with regard to survival (see Kudo et al. (2018) and Janda et al. (2017) among many others). For this purpose an analysis based on Kaplan-Meier curves (Kaplan and Meier 1958), followed by a log-rank test (Kalbfleisch and Prentice 2011) or a modified log-rank test (see, for example, Peto and Peto 1972 and Yang and Prentice 2010) is still the most popular approach. Further, in order to investigate treatment difference over time, simultaneous confidence bands for the difference of two survival curves have been considered (Parzen et al. 1997). Additionally, adjusting for multiple covariates, Cox’s proportional hazards model (Cox 1972) has been extensively used in the last decades (for some examples see Cox and Oakes (1984) and Klein and Moeschberger (2006) among many others). In case of addressing non-inferiority or equivalence, extensions of the log-rank test investigating the vertical distance between the survival curves have been proposed by Wellek (1993) and Com-Nougue et al. (1993). These approaches owe much of their popularity to the fact that they do not rely on assumptions on the distribution of event times. Moreover, a direct interpretation is obtained by summarizing the treatment effect in one single parameter, given by the hazard ratio of the two treatments, assumed to be constant over time.

However, this assumption has been heavily criticized (Hernán 2010; Uno et al. 2014) and is in practice rarely assessed or even obviously violated (Li et al. 2015; Jachno et al. 2019). In particular, if the two treatments’ short- and long-term benefits differ, for instance, when surgical treatment is compared to a non-surgical one (Howard et al. 1997), the assumption of proportional hazards is questionable. The most obvious sign of a violation of this assumption is crossing survival curves. However, graphical methods (Grambsch and Therneau 1994) or statistical tests (Gill and Schumacher 1987) are often needed to detect non-proportional hazards.

One of the advantages of the standard methodology based on Kaplan–Meier curves and the log-rank test is that equivalence hypotheses can be formulated using one parameter, the hazard ratio. If this relationship changes over time, both an alternative measure of treatment effect and an appropriate definition of equivalence must be found. For instance, Royston and Parmar (2011) introduce the restricted mean survival time to overcome this issue. Another non-parametric measure comparing survival times from two groups for right-censored survival data is the Mann–Whitney effect (Dobler and Pauly 2018). Regarding different test procedures, one alternative to commonly used log-rank based tests of equivalence has been proposed by Martinez et al. (2017). These authors show that type I errors for the classical log-rank test are higher than the nominal level if hazards are non-proportional. They present an alternative based on a proportional odds assumption yielding a robust $\alpha$-level test in this setting. In situations where neither hazards nor odds are proportional, Shen (2020) recently proposed an alternative test for equivalence based on a semiparametric log transformation model. Finally, having reviewed the most recent two-armed clinical oncology trials, Dormuth et al. (2022) give a user-friendly overview of which test to use when survival curves cross.

Methods using parametric survival models are less common than the semiparametric or non-parametric methods mentioned above. However, a correctly specified parametric survival model offers numerous advantages, such as more accurate estimates (Klein and Moeschberger 2006) and the ability to make predictions. Inference based on parametric models can be very precise even in case of misspecification, as demonstrated by Subramanian and Zhang (2013), who develop simultaneous confidence bands for parametric survival curves and compare them to non-parametric approaches based on Kaplan–Meier estimates.

In this paper, we develop a new methodology in two directions. We address the issue of non-inferiority and equivalence testing by presenting a parametric alternative to the classical methodology, without assuming proportional hazards. First, we derive pointwise confidence bands for the difference of two survival curves and the hazard ratio over time, by using asymptotic inference and a bootstrap approach. Second, we use these confidence bands to assess equivalence or non-inferiority of two treatments for both pointwise comparisons and for entire time intervals. A similar approach has been proposed by Liu et al. (2009) and Bretz et al. (2018), who derive such confidence bands for assessing the similarity of dose-response curves. Finally, all our methods are illustrated by a clinical trial example and by means of a simulation study, where we also investigate the robustness of our approach.

## Methods

Consider two samples of size $n_1$ and $n_2$ respectively, resulting in a total sample size of $n=n_1+n_2$. Let $Y_{1,1},\ldots ,Y_{1,n_1}$ and $Y_{2,1},\ldots ,Y_{1,n_2}$ denote independent random variables representing survival times for individuals allocated to two (treatment) groups, observing a time range given by ${\mathscr {T}}=\left[ 0, t_{max} \right]$, where 0 denotes the start of the observations and $t_{max}$ a fixed time point of last follow-up. Assume that the distribution functions $F_1$ and $F_2$ of $Y_{1,j},\ j=1,\ldots ,n_1$ and $Y_{2,j}\ j=1,\ldots ,n_2$, respectively, are absolutely continuous with densities $f_1$ resp. $f_2$. Consequently the probability of experiencing an event for an individual *j* of the $\ell$-th sample before time *t* can be written as $F_\ell (t)={\mathbb {P}}(Y_{\ell ,j}< t)=\int _{0}^{t}f_{\ell }(u) du,\ \ell =1,2.$ Further denote the corresponding survival functions by $S_\ell :=1-F_\ell$ and the hazard rates by $h_\ell :=\tfrac{f_\ell }{S_\ell }$. The cumulative hazard function is given by $H_\ell (t)=-\log (S(t))$, $\ell =1,2$.

For the sake of simplicity we do not assume additional covariates. Further, in addition to a fixed end of the study, we assume all observations to be randomly right-censored and denote the censoring times of the two samples by $C_{1,1},\ldots ,C_{1,n_1}$ and $C_{2,1},\ldots ,C_{2,n_2}$ and the corresponding distribution functions by $G_1$ and $G_2$ respectively. Note that these distributions can differ from each other and are assumed to be independent from the $Y_{\ell ,j},\ \ell =1,2,\ j=1,\ldots n_\ell$. We define $\Delta _{\ell ,j}=I\{Y_{\ell ,j} \ge C_{\ell ,j}\},$ indicating whether an individual is censored ($\Delta _{\ell ,j}=0$) or experiences an event ($\Delta _{\ell ,j}=1$), where *I* denotes the indicator function. Consequently the observed data $(t_{\ell ,j},\delta _{\ell ,j})$ is a realization of the bivariate random variable $(T_{\ell ,j},\Delta _{\ell ,j})$, where $T_{\ell ,j}=\min (Y_{\ell ,j},C_{\ell ,j}),\ \ell =1,2,\ j=1,\ldots n_\ell$. In order to make inference on the underlying distributions we consider the likelihood function for group $\ell$ given by

1 $$\begin{aligned}&L_\ell (F_\ell ,G_\ell )= \prod _{j=1}^{n_\ell }\left\{ f_\ell (t_{\ell ,j})^{\delta _{\ell ,j}}(1-F_\ell (t_{\ell ,j}))^{1-\delta _{\ell ,j}}\right\} \nonumber \\&\prod _{j=1}^{n_\ell }\left\{ (1-G_\ell (t_{\ell ,j}))^{\delta _{\ell ,j}}(g_\ell (t_{\ell ,j}))^{1-\delta _{\ell ,j}}\right\} \end{aligned}$$

as censoring times and survival times are assumed to be independent. Hence we can obtain estimates for the densities $f_\ell (t)=f_\ell (t,\theta _\ell )$ of the distributions of the survival times and densities $g_\ell (t)=g_\ell (t,\psi _\ell )$ corresponding to the censoring distributions by deriving the parameters $\hat{\theta }_\ell$ and $\hat{\psi }_\ell$ maximizing $\log {L_\ell }$, $\ell =1,2$. Note that if one is not interested in estimating the underlying distribution of the censoring times, this optimization procedure can be further simplified by just considering the first part in (1), resulting in an objective function given by

2 $$\begin{aligned} {\tilde{L}}_\ell (\theta _\ell )= \prod _{j=1}^{n_\ell }\left\{ f_\ell (t_{\ell ,j},\theta _\ell )^{\delta _{\ell ,j}}(1-F_\ell (t_{\ell ,j},\theta _\ell ))^{1-\delta _{\ell ,j}}\right\} ,\ \ell =1,2, \end{aligned}$$

as $\theta _\ell$ and $\psi _\ell$ have no common parameters.

### Confidence bands

In the following we will construct pointwise confidence bands for the difference of the survival functions and for the hazard ratio. First we derive an asymptotic approach using the Delta-method (Oehlert 1992) and second, we propose an alternative based on a bootstrap procedure. The latter can also be used when samples are very small or if asymptotic inference is impossible due to the lack of an explicit expression for the asymptotic variance of the maximum likelihood estimator (MLE) obtained by maximizing (1) or (2), respectively. In order to simplify calculations, we will consider the log-ratio and therefore the two measures of interest are given by

3 $$\begin{aligned} \Delta (t,\theta _1,\theta _2):=S_1(t,\theta _1)-S_2(t,\theta _2)\text { and } r(t,\theta _1,\theta _2):=\log {\tfrac{h_1(t,\theta _1)}{h_2(t,\theta _2)}}. \end{aligned}$$

Under certain regularity conditions (Bradley and Gart 1962) the MLE $\hat{\theta }_\ell$, $\ell =1,2$, is asymptotically normally distributed. Precisely,

$$\begin{aligned} \sqrt{n_\ell }\big (\hat{\theta }_\ell -\theta _\ell \big ){\mathop {\longrightarrow }\limits ^\mathcal{D}} {\mathscr {N}}(0, {\mathscr {I}}_{\theta _\ell }^{-1}),\ \ell =1,2, \end{aligned}$$

where ${\mathscr {I}}_{\theta _\ell }^{-1}$ denotes the inverse of the Fisher information matrix, $\ell =1,2$. This result can be used to make inference about the asymptotic distribution of the estimated survival curves. Using the Delta-method we obtain for every $t>0$

$$\begin{aligned} \sqrt{n_\ell }\big (S_\ell (t,\hat{\theta }_\ell )-S_\ell (t,\theta _\ell )\big ){\mathop {\longrightarrow }\limits ^\mathcal{D}} {\mathscr {N}}(0, \tfrac{\partial }{\partial \theta _\ell } S_\ell (t,\theta _\ell )^T {\mathscr {I}}_{\theta _\ell }^{-1} \tfrac{\partial }{\partial \theta _\ell } S_\ell (t,\theta _\ell )),\ \ell =1,2. \end{aligned}$$

Consequently, the asymptotic variance of $\Delta (t,\hat{\theta }_1,\hat{\theta }_2)$ is given by

4 $$\begin{aligned} \sigma _\Delta ^2=\tfrac{1}{n_1}\tfrac{\partial }{\partial \theta _1}S_1(t,\theta _1)^T {\mathscr {I}}_{\theta _1}^{-1} \tfrac{\partial }{\partial \theta _1}S_1(t,\theta _1) +\tfrac{1}{n_2}\tfrac{\partial }{\partial \theta _2}S_2(t,\theta _2)^T {\mathscr {I}}_{\theta _2}^{-1} \tfrac{\partial }{\partial \theta _2}S_2(t,\theta _2). \end{aligned}$$

By replacing $\theta _\ell$ by its estimate $\hat{\theta }_\ell$ and ${\mathscr {I}}_{\theta _\ell }^{-1}$ by the observed information matrix $I_{\hat{\theta }_\ell }$, $\ell =1,2$, a consistent estimator $\hat{\sigma }^2_\Delta$ of the asymptotic variance in (4) is obtained (Bradley and Gart 1962). For sufficiently large samples this asymptotic result can be used to construct pointwise lower and upper $(1-\alpha )$-confidence bands, respectively, by

5 $$\begin{aligned} L_\Delta (t,\hat{\theta }_1,\hat{\theta }_2)=\Delta (t,\hat{\theta }_1,\hat{\theta }_2)-z_{1-\alpha } \hat{\sigma }_\Delta \text { and } U_\Delta (t,\hat{\theta }_1,\hat{\theta }_2)=\Delta (t,\hat{\theta }_1,\hat{\theta }_2)+z_{1-\alpha } \hat{\sigma }_\Delta ,\nonumber \\ \end{aligned}$$

where $z_{1-\alpha }$ denotes the $(1-\alpha )$-quantile of the standard normal distribution. More precisely, if *L*(*t*) and *U*(*t*) denote the $(1-\alpha )$ pointwise lower and the $(1-\alpha )$ pointwise upper confidence band, respectively, it holds

6 $$\begin{aligned} \lim _{n_1,n_2\rightarrow \infty }{\mathbb {P}}\big (L_\Delta (t,\hat{\theta }_1,\hat{\theta }_2)\le \Delta (t,\theta _1,\theta _2)\big )&\ge 1-\alpha \text {,}\nonumber \\ \lim _{n_1,n_2\rightarrow \infty }{\mathbb {P}}\big (\Delta (t,\theta _1,\theta _2)\le U_\Delta (t,\hat{\theta }_1,\hat{\theta }_2)\big )&\ge 1-\alpha \end{aligned}$$

for all $t>0$, where $\alpha$ denotes the prespecified significance level. The construction of pointwise confidence bands for the log hazard ratio is done similarly, and an estimate of the asymptotic variance of $r(t,\hat{\theta }_1,\hat{\theta }_2)$ is given by

7 $$\begin{aligned} \hat{\sigma }_r^2=\,&\tfrac{1}{n_1}\cdot \tfrac{\partial }{\partial \theta _1}\log {h_1(t,\hat{\theta }_1)}^T I_{\theta _1}^{-1} \tfrac{\partial }{\partial \theta _1}\log {h_1(t,\hat{\theta }_1)}\nonumber \\&+ \tfrac{1}{n_2}\cdot \tfrac{\partial }{\partial \theta _2}\log {h_2(t,\hat{\theta }_2)}^T I_{\theta _2}^{-1} \tfrac{\partial }{\partial \theta _2} \log {h_2(t,\hat{\theta }_2)}. \end{aligned}$$

Consequently $L_r$ and $U_r$ are given by

8 $$\begin{aligned} L_r(t,\hat{\theta }_1,\hat{\theta }_2)=r(t,\hat{\theta }_1,\hat{\theta }_2)-z_{1-\alpha } \hat{\sigma }_r \text {, } U_r(t,\hat{\theta }_1,\hat{\theta }_2)=r(t,\hat{\theta }_1,\hat{\theta }_2) +z_{1-\alpha }\hat{\sigma }_r. \end{aligned}$$

A concrete numerical example for calculating the estimates $\hat{\sigma }_{\Delta }^2$ and $\hat{\sigma }_r^2$ and the corresponding confidence bands assuming a Weibull distribution are deferred to Section A2 of the Appendix. If sample sizes are rather small or the variability in the data is high, we propose to obtain estimates for the variances $\hat{\sigma }_{\Delta }^2$ and $\hat{\sigma }_r^2$ by using a bootstrap approach, taking the right-censoring into account. This method can also be used if a formula for the asymptotic variance is not obtainable, for instance due to numerical difficulties. The following algorithm explains the procedure for $\Delta (t_0,\theta _1,\theta _2)$ and it can directly be adapted to $r(t_0,\theta _1,\theta _2)$.

#### Algorithm 1

(Parametric) Bootstrap Confidence Bands for $\Delta (t_0,\theta _1,\theta _2)$.

- Calculate the MLE $\hat{\theta }_\ell$ and $\hat{\psi }_\ell ,\ \ell =1,2$, from the data by maximizing (1).
- Generate survival times $y_{\ell ,1}^*,\ldots ,y_{\ell ,n_\ell }^*$ from $F_\ell (\hat{\theta }_\ell )$, $\ell =1,2$. Further generate the corresponding censoring times $c_{\ell ,1}^*,\ldots ,c_{\ell ,n_\ell }^*$ by sampling from the distributions $G_\ell (\hat{\psi }_\ell )$, $\ell =1,2$. If $y_{\ell ,j}^*>c_{\ell ,j}^*$, the observation is censored (i.e. $\delta _{\ell ,j}^*=0$), $j=1,\ldots ,n_\ell$. The observed data is given by $(t_{\ell ,j}^*,\delta _{\ell ,j}^*)$, $t_{\ell ,j}^*=min(y_{\ell ,j}^*,c_{\ell ,j}^*)$, $j=1,\ldots ,n_\ell$, $\ell =1,2$.
- Calculate MLE $\hat{\theta }_\ell ^*$ for the bootstrap sample from the $t_{\ell ,j}^*,\ j=1,\ldots ,n_\ell ,\ \ell =1,2$, and calculate the difference of the corresponding survival functions at $t_0$, that is
  9 $$\begin{aligned} \Delta ^*:=\Delta (t_0,\hat{\theta }_1^*,\hat{\theta }_2^*)=S_1(t_0,\hat{\theta }_1^*)-S_2(t_0,\hat{\theta }_2^*). \end{aligned}$$
- Repeating steps 2*a* and 2*b* $n_{boot}$ times yields $\Delta ^*_1,\ldots , \Delta ^*_{n_{boot}}$. Calculate an estimate for the variance $\hat{\sigma }_{\Delta }^2$ by
  10 $$\begin{aligned} \hat{\sigma }_{\Delta }^2=\frac{1}{n_{boot}-1}\sum _{k=1}^{n_{boot}}(\Delta ^*_k-\bar{\Delta }^*)^2, \end{aligned}$$
  where $\bar{\Delta }^*$ denotes the mean of the $\Delta _i^*,\ i=1,\ldots ,n_{boot}$.

Finally the estimate $\hat{\sigma }_{\Delta }^2$ in (10) is used to calculate the confidence band in (5). The procedure described in Algorithm 1 is a parametric bootstrap based on estimating the parameters $\hat{\theta }_\ell ,\ \hat{\psi }_\ell ,\ \ell =1,2$. A non-parametric alternative, given by resampling the observations, could also be implemented (Efron 1981; Akritas 1986). However, it has been shown that the parametric bootstrap tends to be more accurate if the underlying parametric model is correctly specified (Efron and Tibshirani 1994). Note that the asymptotic inference approach to obtaining confidence bands does not require estimating the censoring distributions. Consequently, the MLE $\hat{\theta }_\ell$ can be obtained by maximizing the likelihood function ${\tilde{L}}_\ell$ in Eq. (2), $\ell =1,2$. On the other hand, the bootstrap proposed in Algorithm 1 requires additional estimation of the censoring distributions and therefore requires the more involved maximization of Eq. (1).

### Equivalence and non-inferiority tests

We are aiming to compare the survival functions of two (treatment) groups which is commonly addressed by testing the null hypothesis that the two survival functions are identical against the alternative hypothesis that the survival functions differ at least at a single time point (for a review, see, e.g., Klein and Moeschberger 2006). More precisely the classical hypotheses are given by

$$\begin{aligned} H_{0} :S_{1} (t,\theta _{1} ) = S_{2} (t,\theta _{2} ){\text { for all }}t \in \mathscr {T} {\text { against }}H_{1} :S_{1} (t,\theta _{1} ) \ne S_{2} (t,\theta _{2} ){\text { for}}\;{\text {a}}\;t \in \mathscr {T}. \end{aligned}$$

Sometimes one is more interested in observing the non-inferiority of one treatment to another or the equivalence of the two treatments, meaning that we allow a deviation of the survival curves of a prespecified threshold instead of testing for equality. This can be done for a particular point in time or over an entire interval, for example the whole observational period.

#### Comparing survival for one particular point in time

We start by considering the difference in survival at a particular point in time $t_0$. The corresponding hypotheses are then given by

11 $$\begin{aligned} H_0: S_1(t_0,\theta _1)-S_2(t_0,\theta _2)\ge \delta \text { against } H_1:S_1(t_0,\theta _1)-S_2(t_0,\theta _2)< \delta \end{aligned}$$

for a non-inferiority trial observing whether a test treatment is non-inferior to the reference treatment (which is stated in the alternative hypothesis). Considering equivalence, we test

12 $$\begin{aligned} H_0: \left| S_1(t_0,\theta _1)-S_2(t_0,\theta _2)\right| \ge \delta \text { against } H_1: \left| S_1(t_0,\theta _1)-S_2(t_0,\theta _2)\right| < \delta . \end{aligned}$$

The same questions can be addressed considering the (log) hazard ratio, resulting in the hypotheses analogue to (11) given by

13 $$\begin{aligned} H_0: \log {\tfrac{h_1(t_0,\theta _1)}{h_2(t_0,\theta _2)}}\ge \epsilon \text { against } H_1: \log {\tfrac{h_1(t_0,\theta _1)}{h_2(t_0,\theta _2)}}< \varepsilon \end{aligned}$$

for a non-inferiority trial and

14 $$\begin{aligned} H_0: \left| \log {\tfrac{h_1(t_0,\theta _1)}{h_2(t_0,\theta _2)}}\right| \ge \epsilon \text { against } H_1: \left| \log {\tfrac{h_1(t_0,\theta _1)}{h_2(t_0,\theta _2)}}\right| < \varepsilon \end{aligned}$$

for addressing equivalence. The choice of the margins $\delta >0$ and $\epsilon >0$ has to be verified in advance with great care combining statistical and clinical expertise. From a regulatory point of view there is no fixed rule but general advice can be found in a guideline of the EMA (2014). Following recent literature, margins $\delta$ for the survival difference are frequently chosen between 0.1 and 0.2 (D’Agostino Sr et al. 2003; Da Silva et al. 2009; Wellek 2010).

In the following, we will only consider the hypotheses in Eqs. (11) and (12) referring to the difference of the survival curves; a similar procedure can be applied for testing the hypotheses in Eqs. (13) and (14), respectively. Therefore, we use the confidence bands derived in Eq. (5) for defining an asymptotic $\alpha$-level test for (11). The null hypothesis in Eq. (11) is rejected and non-inferiority is claimed if the upper bound of the confidence band is below the margin, that is

15 $$\begin{aligned} U_\Delta (t_0,\hat{\theta }_1,\hat{\theta }_2)\le \delta . \end{aligned}$$

Further, an equivalence test for the hypotheses in Eq. (12) is defined by rejecting $H_0$ whenever

16 $$\begin{aligned} U_\Delta (t_0,\hat{\theta }_1,\hat{\theta }_2)\le \delta \text { and } L_\Delta (t_0,\hat{\theta }_1,\hat{\theta }_2)\ge -\delta . \end{aligned}$$

Note that according to the intersection–union-principle (Berger 1982), the $(1-\alpha )$-confidence bands $L_\Delta (t_0,\hat{\theta }_1,\hat{\theta }_2)$ and $U_\Delta (t_0,\hat{\theta }_1,\hat{\theta }_2)$ are used for both, the non-inferiority and the equivalence test. The following lemma states that this yields an asymptotic $\alpha$-level test.

##### Lemma 1

The test described in Eq. (16) yields an asymptotic $\alpha$-level equivalence tests for the hypotheses in Eq. (12). More precisely it holds for all $t_0\in \mathcal T$

$$\begin{aligned} \lim _{n_1,n_2\rightarrow \infty }{\mathbb {P}}_{H_0}(U_\Delta (t_0,\hat{\theta }_1,\hat{\theta }_2)\le \delta , L_\Delta (t_0,\hat{\theta }_1,\hat{\theta }_2)\ge -\delta )\le \alpha . \end{aligned}$$

The proof is left to Section A1 of the Appendix.

#### Comparing survival over an entire period of time

From a practical point of view there are situations where it might be interesting to compare survival not only at one particular point in time but over an entire period of time $[t_1,t_2]$, which could also be the entire observation period $\mathcal T$. This means that for instance the null hypothesis in Eq. (12) is extended to investigating $\left| S_1(t,\theta _1)-S_2(t,\theta _2)\right| \ge \delta$ for all *t* in $[t_1,t_2]$. This yields the hypotheses

17 $$\begin{aligned} {\tilde{H}}_0: \max _{t\in [t_1,t_2]}\left| S_1(t,\theta _1)-S_2(t,\theta _2)\right| \ge \delta \text { against } {\tilde{H}}_1 : \max _{t\in [t_1,t_2]} \left| S_1(t,\theta _1)-S_2(t,\theta _2)\right| < \delta \end{aligned}$$

and a similar extension can be formulated for the non-inferiority test (11) and the tests on the hazard ratio stated in Eqs. (13) and (14), respectively. In this case we conduct a test as defined in Eq. (16) on each time point in the observational period and reject the null hypothesis in Eq. (17) if each pointwise null hypothesis as stated in (12) is rejected. Consequently, this means that the null hypothesis $\tilde{H}_0$ in Eq. (17) is rejected if for all *t* in $[t_1,t_2]$, the confidence bands $L_\Delta (t,\hat{\theta }_1,\hat{\theta }_2)$ and $U_\Delta (t,\hat{\theta }_1,\hat{\theta }_2)$ derived in (5) are included in the equivalence region $\left[ -\delta ,\delta \right]$, which can be also formulated as

18 $$\begin{aligned} -\delta \le \min _{t\in [t_1,t_2]} L_\Delta (t,\hat{\theta }_1,\hat{\theta }_2) \text { and } \max _{t\in [t_1,t_2]} U_\Delta (t,\hat{\theta }_1,\hat{\theta }_2) \le \delta . \end{aligned}$$

In order to prove that this yields an asymptotic $\alpha$-level test, we first note that the rejection region of ${\tilde{H}}_0$ in Eq. (17) is the intersection of the rejection regions of the two sub-hypotheses ${\tilde{H}}_0^1: \max _{t\in [t_1,t_2]} S_1(t,\theta _1)-S_2(t,\theta _2)\ge \delta$ and $\tilde{H}_0^2: \min _{t\in [t_1,t_2]} S_1(t,\theta _1)-S_2(t,\theta _2)\le -\delta$. Again, due to the intersection–union-principle (Berger 1982), it is therefore sufficient to show that each of these individual tests is an asymptotic $\alpha$-level test. Without loss of generality we consider the non-inferiority test, where we reject the null hypothesis ${\tilde{H}}_0^1$ if $\max _{t\in [t_1,t_2]} U_\Delta (t,\hat{\theta }_1,\hat{\theta }_2) \le \delta$. Denoting by ${\tilde{t}}$ the point in $[t_1,t_2]$ with $\max _{t\in [t_1,t_2]} \Delta (t,\theta _1,\theta _2)=\Delta ({\tilde{t}},\theta _1,\theta _2)$ yields

$$\begin{aligned}&{\mathbb {P}}_{{\tilde{H}}_0^1}(\max _{t\in [t_1,t_2]} U_\Delta (t,\hat{\theta }_1,\hat{\theta }_2) \le \delta )\\&\qquad = {\mathbb {P}}_{{\tilde{H}}_0^1}(\max _{t\in [t_1,t_2]} U_\Delta (t,\hat{\theta }_1,\hat{\theta }_2) - \Delta (\tilde{t},\theta _1,\theta _2) \le \delta -\Delta (\tilde{t},\theta _1,\theta _2))\\&\qquad \le {\mathbb {P}}(\max _{t\in [t_1,t_2]} U_\Delta (t,\hat{\theta }_1,\hat{\theta }_2)\le \Delta (\tilde{t},\theta _1,\theta _2)) \end{aligned}$$

and as $\max _{t\in [t_1,t_2]} U_\Delta (t,\hat{\theta }_1,\hat{\theta }_2)\ge U_\Delta (\tilde{t},\hat{\theta }_1,\hat{\theta }_2)$ the assertion follows with (6).

Of note, this result also implies that the construction of simultaneous confidence bands, which are wider than pointwise ones and hence would result in a more conservative test, is not necessary.

## Finite sample properties

In the following we will investigate the finite sample properties of the proposed methods by means of a simulation study. Survival times are distributed according to a Weibull distribution and a log-logistic distribution, respectively, where the latter scenario will be used for investigations on the robustness of the approach. We assume (randomly) right-censored observations in combination with an administrative censoring time in both scenarios. All results are obtained by running $n_{sim}=1000$ simulations and $n_{boot}=500$ bootstrap repetitions. For all three scenarios we will calculate confidence bands for both the difference of the survival curves and the log hazard ratio and observe their coverage probabilities. For the difference of the survival curves, we will investigate the tests on non-inferiority and equivalence proposed in Eqs. (15), (16) and (18), respectively. For this purpose we will vary both, the particular time point under consideration $t_0$ and the non-inferiority/equivalence margin $\delta$. More precisely we will consider three different choices for this margin, namely $\delta =0.1,0.15$ and 0.2. Additionally we also evaluate all scenarios using a non-parametric approach as described in Sect. 5.2. of Com-Nougue et al. (1993). Precisely we construct confidence bands for the difference of two Kaplan–Meier curves by estimating the variance using Greenwood’s formula (Greenwood 1926). This approach also comes along without the assumption of proportional hazards and consequently it can be directly compared to our method, which will be referred to as "the parametric approach" in the following. Further, we investigate the performance of the test when comparing survival over the entire observational period (that is ${\mathscr {T}}=[0,t_{max}]$) as described in Sect. 2.2.2. Due to the sake of brevity, the detailed results for this analysis are deferred to Section 4 of the Supplementary Material.

**Fig. 1:**
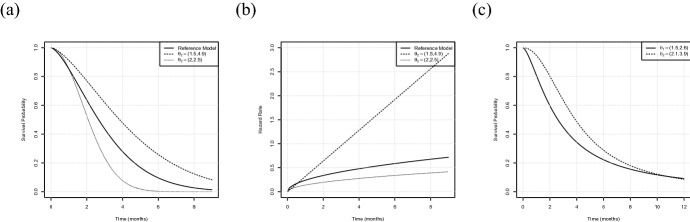
The three scenarios under consideration used for simulating type I error rates and coverage probabilities **a** Survival curves for Scenarios (19) and (20) with $\theta _1=(1.5,3.4)$, $\theta _2\in \left\{ (1.5,4.9),(2,2.5)\right\}$. **b** Corresponding hazard rates. **c** Survival curves for Scenario (21)

**Fig. 2:**
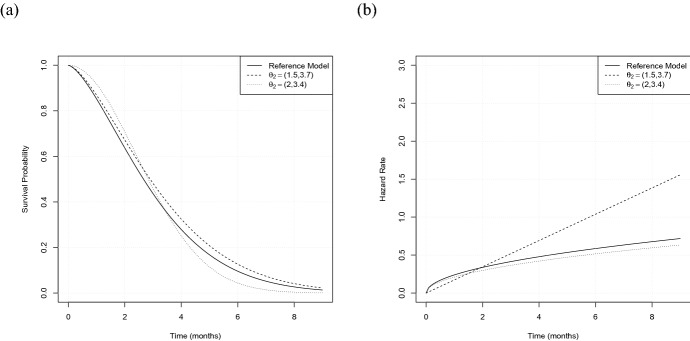
The two scenarios used for simulating the power. **a** Survival curves for Scenarios (19) and (20) with $\theta _1=(1.5,3.4)$, $\theta _2\in \left\{ (1.5,3.7),(2,3.4)\right\}$. **b** Corresponding hazard rates

For the first two scenarios we assume the data in both treatment groups to follow a Weibull distribution, that is $F_\ell (t,\theta _\ell )=1-\exp {\left\{ -(t/\theta _{\ell ,2})^{\theta _{\ell ,1}}\right\} }$, $\ell =1,2$, where $\theta _{\ell ,1}$ denotes the shape parameter and $\theta _{\ell ,2}$ the scale parameter corresponding to treatment group $\ell =1,2$. We consider a time range (in months) given by ${\mathscr {T}}=[0,9]$, where $t_{max}=9$ is the latest point of follow up. For the first configuration we choose

19 $$\begin{aligned} \theta _1=(1.5,3.4),\ \theta _2^1=(1.5,4.9),\ \theta _2^2=(1.5,3.7), \end{aligned}$$

where $\theta _1$ corresponds to the reference model and the second model is varied by its scale parameter. Here $\theta _2^1$ is used for investigating the type I errors and coverage probabilities and $\theta _2^2$ for simulating the power, respectively (see Figs. 1 and 2). As an example, Fig. 1a displays the survival curves for a choice of $\theta _2^1$. Both configurations result in a constant log hazard ratio of $\log {(1.5)}\approx 0.4$, representing the situation of proportional hazards. We assume the censoring times to be exponentially distributed and choose the rates of the two groups such that a censoring rate of approximately $25\%$ results, that is a rate of $\psi _1=0.1$ for the reference model and rates of $\psi _2^1=0.09$ and $\psi _2^2=0.05$ for $\theta _2^1$ and $\theta _2^2$, respectively. In order to investigate the effect of non-proportional hazards we consider a second scenario of intersecting survival curves, where we keep the reference model specified by $\theta _1$ and all other configurations as above, but vary the parameters of the second model, resulting in

20 $$\begin{aligned} \theta _1=(1.5,3.4),\ \theta _2^1=(2,2.5),\ \theta _2^2=(2,3.4). \end{aligned}$$

Here the choice of $\theta _2^1$ is used for investigating the type I errors and coverage probabilities and $\theta _2^2$ for simulating the power, respectively. Again, we consider censoring rates of approximately $25\%$ for both treatment groups, meaning a rate of $\psi _1^1=0.14$ and $\psi _2^1=0.1$ for $\theta _2^1$ and $\theta _2^2$, respectively. In the following scenario (19) is denoted as “PH” (proportional hazards) and scenario (20) as “NPH” (non-proportional hazards).

In order to investigate the effect of different censoring rates on both procedures, we additionally consider the NPH Scenario (20) with a fixed sample size of $(n_1,n_2)=(100,100)$ but vary the parameters $\psi _\ell ,\ \ell =1,2$, such that given the latest time point of follow-up by $t_{max}=9$, between 10 and $75\%$ of the individuals are censored. Precisely, we also consider unbalanced situations where censoring rates are different across the two groups. The results are presented and discussed in Sect. 2 of the Supplementary Material.

Afterwards, we will analyze the robustness of the approach in two different ways. First, we will use the NPH scenario (20) in order to investigate the effect of misspecifying the distribution of the censoring times. Precisely, we will assume the censoring times to follow a uniform distribution instead of the true underlying exponential distribution. Note that this only affects the bootstrap-based confidence bands described in Algorithm 1 as the asymptotic bands do not require any estimation of the underlying censoring distribution. The detailed results of this analysis will be deferred to Sect. 1 of the Supplementary Material, we will briefly summarize them in Sect. 3.1. For further robustness investigations, we consider a third scenario, where we generate survival times according to a log-logistic distribution. Precisely we choose $F_\ell (t,\theta _\ell )=1-\tfrac{1}{1+(t/\theta _{\ell ,1})^{-\theta _{\ell ,2}}}$, $\ell =1,2$. We now generate censoring times according to a uniform distribution on an interval $[0,c_\ell ]$, where $c_\ell$ is chosen such that a censoring rate of approximately $20\%$ results, $\ell =1,2$. We consider a time range (in months) given by $\mathscr {T}=[0,12]$ and define the scenario by the set of parameters given by

21 $$\begin{aligned} \theta _1=(1.5,2.6),\ \theta _2=(2.1,3.9), \end{aligned}$$

where $\theta _1$ corresponds to the reference model, see Fig. 1c. We will use this configuration to investigate the performance of the proposed method under the situation of misspecification of the event times. Precisely, we assume that the event times follow a Weibull distribution instead of the log-logistic distribution. The results will be presented in Sects. 3.1 and 3.2 and compared to the non-parametric approach.

### Coverage probabilities

In order to investigate the performance of the confidence bands derived in Eqs. (5) and (8) we consider the scenarios described above for three different sample sizes, that is $(n_1,n_2)=(20,20)$, $(n_1,n_2)=(50,50)$ and $(n_1,n_2)=(100,100)$, resulting in total sample sizes given by $n=40,\ 100$ and 200, respectively. We choose a nominal level of $\alpha =0.05$ and calculate both, the asymptotic (two-sided) confidence bands obtained by using the Delta method, and the bands based on the bootstrap described in Algorithm 1, which we call in the following asymptotic bands and bootstrap bands, respectively. All bands were constructed for an equidistant grid of 23 time points ranging from 1.5 to 6 months for the first scenario and for a grid of 14 different points ranging from 1.5 to 4 months for the second one, respectively. For the investigations on the situation of misspecification we consider 21 time points, ranging from 1 to 5 months.

**Fig. 3:**
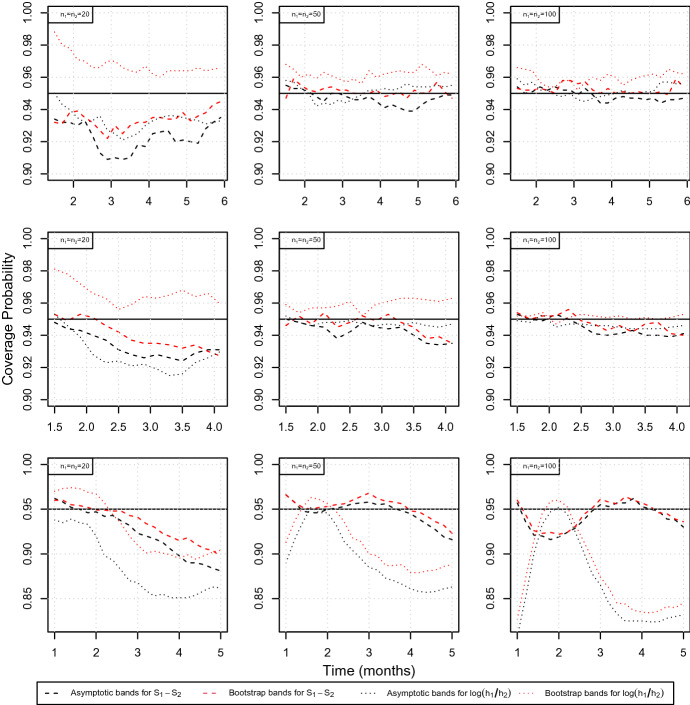
Simulated coverage probabilities for the PH scenario (19) (first row), the NPH scenario (20) (second row) and the scenario of misspecification (21) (third row) at different time points for sample sizes of $n_1=n_2=20,\ 50,\ 100$ (left, middle, right column). The dashed lines correspond to the confidence bands for the difference of the survival functions (5), the dotted lines to the confidence bands for the log hazard ratio (8). Black lines display the asymptotic bands, red lines the confidence bands based on bootstrap, respectively

We first consider the two correctly specified scenarios (event distribution of reference and test group is Weibull and modelled as such). For the first configuration the hazard ratio is constant over time, for the second it varies between 0.5 and 2 on the grid described above, or, equivalently, from $-0.6$ to 0.7 considering the log hazard ratio. The first two rows of Fig. 3 summarize the simulated coverage probabilities for these scenarios. In general it turns out that for both scenarios and all approaches, i.e. the asymptotic bands and the bootstrap bands for $S_1-S_2$ and $\log {\tfrac{h_1}{h_2}}$, respectively, the approximation is very precise when sample sizes increase, as the coverage probabilities are very close to the desired value of 0.95 in this case. Further it becomes obvious that the confidence bands obtained by estimating the variance by bootstrap (10) are always slightly more conservative than their asymptotic versions (4) and (7), respectively.

However, considering the bands on $S_1-S_2$ for very small sample sizes, that is $n_1=n_2=20$, the coverage probability lies between 0.91 and 0.94 and hence these bands are rather liberal. The bootstrap bands perform slightly better, but still have coverage probabilities around 0.93 instead of 0.95, see the first column of Fig. 3. This effect already disappears for $n_1=n_2=50$ where a highly improved accuracy can be observed. The asymptotic bands for $\log {\tfrac{h_1}{h_2}}$ perform similarly, whereas the bootstrap bands show a different behaviour, that is being rather conservative for small sample sizes, but also getting more precise with increasing sample sizes. For smaller sample sizes, all confidence bands under consideration vary in their behaviour over time. This effect gets in particular visible when considering the NPH scenario (20), see the second row of Fig. 3. The coverage probabilities of the bands for $S_1-S_2$ start with a very accurate approximation during the first two months but then decrease to 0.93. This effect can be explained by the fact that in the setting of a very small sample, that is $n_1=n_2=20$, after this period only very few patients remain (note that the median survival for the reference model is given by 2.6 months) and hence the uncertainty in estimating the variance increases. The same holds for all bands under consideration, explaining the decreasing accuracy at later time points.

We further investigated the effect of misspecifying the censoring distribution. Of note, this misspecification does only affect the bootstrap bands and not the asymptotic confidence bands, as these do not take the censoring mechanism into account. To this end, we considered the NPH scenario (20) and assumed a uniform distribution of the censoring times instead of the true underlying exponential distribution. A figure showing the coverage probabilities compared to the correctly specified situation is deferred to the Supplementary Material. It turns out that the effect of misspecification is rather small. The bands tend to be slightly more conservative as the coverage probabilities are close to 1 for the small sample size setting of $n_1=n_2=20$. However, in general they are above the desired value of 0.95 in all scenarios and for increasing sample sizes this approximation is very precise. We therefore conclude that the bootstrap bands are very robust against misspecification of the censoring distribution.

Finally, we consider the log-logistic scenario of misspecification (21), where we erroneously assumed a Weibull distribution instead of the underlying log-logistic distribution. Further, concerning the bootstrap approach, the censoring distribution was assumed to be exponential and hence misspecified as well. In this scenario the hazard ratio varies from 2.5 to 0.8 over a time from 1 to 5 months, meaning that hazards are non-proportional. The corresponding coverage probabilities are shown in the third row of Fig. 3. It turns out that the performance is worse than in case of a correctly identified model as the coverage lies between 0.85 and 0.9 and hence below the desired value of 0.95. However, considering $S_1-S_2$, in a majority of the cases the coverage is above 0.9, even for a small sample size of $n=40$, where the bootstrap approach performs slightly better than the asymptotic analogue despite of suffering from an additional missspecification issue due to the censoring assumption. This result is in line with our findings from the NPH scenario (20), demonstrating that the bootstrap confidence bands are very robust against misspecifying the censoring distribution. For increasing sample sizes the coverage, which varies over the time, approximates 0.95, whereas the bands for $\log {\tfrac{h_1}{h_2}}$ still do not come sufficiently close to this desired value, even for the largest sample size of $n=200$. Consequently we conclude that these bands suffer more from misspecification than the ones for $S_1-S_2$, where the latter prove to be robust if sample sizes are sufficiently large.

### Type I errors

In the following we will first investigate type I error rates for the non-inferiority test (15) and the equivalence test (16) on the pointwise difference of survival curves. Secondly we will consider entire time intervals, as described in (18). We set $\alpha =0.05$ and consider different sample sizes, i.e. $n_1=n_2\in \{20,50,100,150,250\}$, resulting in total sample sizes given by $n=40,100,200,300$ and 500, respectively. As already indicated by the simulated coverage probability presented in Fig. 3 the difference between asymptotic and bootstrap based bands is very small, in particular for total sample sizes larger than 50. This also holds for the test and hence, for the sake of brevity, we only display the results for the asymptotic version here.

**Fig. 4:**
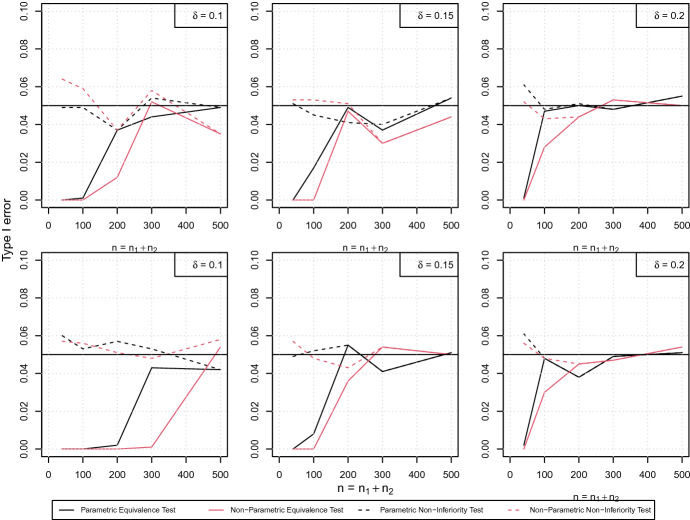
Simulated type I errors for the PH scenario (19) (first row) and the NPH scenario (20) (second row) depending on the sample size. Type I errors have been simulated on the margin of the null hypothesis, that is $\left| S_2(t_0)-S_1(t_0)\right| =\delta =0.1,\ 0.15,\ 0.2$ (left, middle, right column). The dashed lines correspond to the non-inferiority test, the solid lines to the equivalence test. Black lines display the new, parametric approach ((15), (16)), red lines the non-parametric method (Color figure online)

We start with the PH scenario (19) and choose $\theta _2=(1.5,4.9)$, such that the difference curve $S_2(t)-S_1(t)$ attains values of 0.1, 0.15 and 0.2 at time points 1.6, 2.3 and 4, respectively, see Fig. 1a. The median survival is given by 3.8 months and 2.7 months, respectively. The first row of Fig. 4 displays the type I errors simulated on the margin of the null hypothesis for every choice of $\delta$ for both, the non-inferiority tests (dashed lines) and the equivalence tests (solid lines). It turns out that the approximation of the level is very precise, for the non-inferiority test (15) in general and for the equivalence test (16) for sufficiently large sample sizes, as the type I errors are very close to 0.05 in these cases. For small samples, that is $n=n_1+n_2<100$, the equivalence test (16) is conservative as the obtained type I errors are close to zero. The same conclusions can be drawn for the non-parametric approach (red lines). Again, the non-inferiority test approximates the significance level very precisely for all scenarios under consideration, whereas the equivalence test is conservative, but gets more precise with increasing sample sizes. Similar arguments hold for the NPH scenario (20) with $\theta _2=(2,2.5)$, see the second row of Fig. 4. All results obtained here are qualitatively the same as the ones for the PH scenario (19), demonstrating that the presence of a non-constant hazard ratio does not affect the performance of the test. In general, for all procedures the most precise approximation of the significance level is obtained for a large equivalence/non-inferiority margin of $\delta =0.2$. For situations, where the (absolute) difference of the survival curves is even larger than $\delta$, the type I errors are practically zero for all configurations. The corresponding tables, which also include the numbers visualized in Fig. 4, are deferred to the Supplementary Material, Section 3.

Finally, we used the log-logistic scenario (21) to investigate the robustness of the approach regarding the type I error. It turns out that the robustness depends on the equivalence/non-inferiority margin. For the large margin $\delta =0.2$ and for some few configurations of $\delta =0.15$ we observe a low to moderate type I error inflation, whereas for $\delta =0.1$ there is no single type I error above its nominal level. Of note, these results depend on the chosen scenario, i.e. the true underlying models and the time points under consideration. As expected, the non-parametric approach does not suffer from this misspecification issue, which is a direct consequence of its construction with no need of assuming a particular distribution. We conclude that the correct specification of the underlying distribution is very important for the performance of the parametric approach. Although the choice of the equivalence/non-inferiority margin should clearly be determined by practical considerations rather than statistical properties, it should be noted that the risk of a type I error can be reduced by choosing a conservative, i.e. smaller, margin. The detailed tables presenting the simulation results can be found in Section 1 of the Supplementary Material.

### Power

**Fig. 5:**
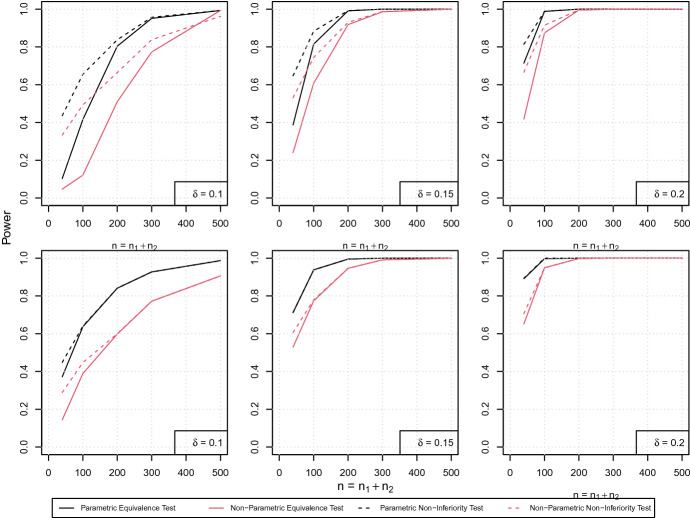
Simulated power for the PH scenario (19) at $(t_0,S_2(t_0)-S_1(t_0))=(0.7,0.01)$ (first row) and the NPH scenario (20) at $(t_0,S_1(t_0)-S_2(t_0))=(0.6,0.04)$ (second row) depending on the sample size for different non-inferiority/equivalence margins $\delta =0.1,\ 0.15,\ 0.2$ (left, middle, right column). The dashed lines correspond to the non-inferiority test, the solid lines to the equivalence test. Black lines display the new, parametric approach ((15), (16)), red lines the non-parametric method (Color figure online)

For investigations on the power we consider the same configurations as given above. We now observe the PH scenario (19) such that the difference curve $S_1(t)-S_2(t)$ attains values of 0.01, 0.02 and 0.04 at time points 0.7, 1.2 and 2.3, respectively. Hence all chosen configurations belong to the alternatives in (15) and (16). For the NPH scenario (20) we consider $\theta _2=(2,3.4)$, resulting in differences of 0.01 and 0.04, attained at time points 0.2 and 0.6 (0.01) and 3.2 and 2.7 (0.04), respectively (see Fig. 2). Figure 5 visualizes the power of both scenarios in dependence of the sample size. Therefore we chose two specific configurations, that is $(t_0,S_2(t_0)-S_1(t_0))=(0.7,0.01)$ for the PH scenario (19) (first row) and $(t_0,S_2(t_0)-S_1(t_0))=(0.6,0.04)$ for the NPH scenario (20) (second row). It becomes obvious that in general the power of all tests clearly increases with increasing sample sizes and a wider equivalence/non-inferiority margin $\delta$. For instance, when considering $\delta =0.2$ the maximum power is close to or larger than $80\%$ for all sample sizes and both tests. In general, the power of the parametric approach is higher than for the non-parametric method for all configurations. Of note, considering a medium threshold of $\delta =0.15$, both tests have a power of approximately 1 if the sample size is sufficiently large, i.e. $n=n_1+n_2>300$. However, for smaller sample sizes or a smaller margin $\delta$ the parametric approach provides a power benefit of up to 0.2 which underlines the theoretical findings.

Considering different time points, the results for the PH scenario (19) can be found in Table 1 for the parametric approach and in Table 2 for the non-parametric approach, respectively. Similarly, Tables 3 and 4 display the power of the two methods in case of the NPH scenario (20), taking four different time points into consideration. For the latter, it becomes obvious that for later time points but equal distances between the two survival curves the power decreases, in particular in presence of small sample sizes. This can be explained by the fact that the remaining subjects become less with progressing time, resulting in a higher uncertainty after 3 months compared to 0.2 and 0.6 months, respectively. Of note, at 3 months more than half of the subjects experienced an event in this scenario. Again, comparing the results of the two approaches demonstrates a clear superiority of the parametric approach if sample sizes are small. For example, considering the PH scenario (19) with $\delta =0.1$ and a sample size of $(n_1,n_2)=(50,50)$ we observe a maximum power of 0.121 for the equivalence test based on the non-parametric approach whereas it is 0.416 for the new parametric approach.

**Table 1:** Simulated power of the non-inferiority test (15) (numbers in brackets) and the equivalence test (16) for the PH scenario (19) with $\theta _2=(1.5,3.7)$ at three different time points $t_0=0.7,1.2,2.3$ for different sample sizes and equivalence margins $\delta$. The nominal level is chosen as $\alpha =0.05$

$(n_1,n_2)$	$(t_0,S_2(t_0)-S_1(t_0))$	$\delta =0.1$	$\delta =0.15$	$\delta =0.2$
(20, 20)	(0.7, 0.01)	0.103 (0.436)	0.387 (0.647)	0.714 (0.814)
	(1.2, 0.02)	0.002 (0.242)	0.019 (0.370)	0.191 (0.560)
	(2.3, 0.04)	0.000 (0.133)	0.000 (0.187)	0.000 (0.263)
(50, 50)	(0.7, 0.01)	0.416 (0.655)	0.855 (0.883)	0.988 (0.988)
	(1.2, 0.02)	0.004 (0.344)	0.371 (0.584)	0.716 (0.793)
	(2.3, 0.04)	0.000 (0.153)	0.000 (0.236)	0.149 (0.415)
(100, 100)	(0.7, 0.01)	0.804 (0.838)	0.991 (0.992)	1.000 (1.000)
	(1.2, 0.02)	0.265 (0.473)	0.787 (0.822)	0.979 (0.980)
	(2.3, 0.04)	0.000 (0.190)	0.240 (0.429)	0.582 (0.652)
(150, 150)	(0.7, 0.01)	0.951 (0.957)	1.000 (1.000)	1.000 (1.000)
	(1.2, 0.02)	0.510 (0.619)	0.940 (0.942)	0.999 (0.999)
	(2.3, 0.04)	0.004 (0.249)	0.460 (0.527)	0.818 (0.827)
(250, 250)	(0.7, 0.01)	0.993 (0.993)	1.000 (1.000)	1.000 (1.000)
	(1.2, 0.02)	0.803 (0.820)	0.989 (0.989)	1.000 (1.000)
	(2.3, 0.04)	0.272 (0.375)	0.734 (0.743)	0.942 (0.942)

**Table 2:** Simulated power of the non-parametric approach for the non-inferiority test (numbers in brackets) and the equivalence test for the PH scenario (19) with $\theta _2=(1.5,3.7)$ at three different time points for different sample sizes and equivalence margins. The nominal level is chosen as $\alpha =0.05$

$(n_1,n_2)$	$(t_0,S_2(t_0)-S_1(t_0))$	$\delta =0.1$	$\delta =0.15$	$\delta =0.2$
(20, 20)	(0.7, 0.01)	0.047 (0.333)	0.240 (0.531)	0.418 (0.666)
	(1.2, 0.02)	0.001 (0.204)	0.021 (0.303)	0.054 (0.429)
	(2.3, 0.04)	0.000 (0.119)	0.000 (0.197)	0.000 (0.282)
(50, 50)	(0.7, 0.01)	0.121 (0.493)	0.608 (0.744)	0.874 (0.915)
	(1.2, 0.02)	0.002 (0.264)	0.106 (0.435)	0.456 (0.643)
	(2.3, 0.04)	0.000 (0.160)	0.000 (0.210)	0.007 (0.312)
(100, 100)	(0.7, 0.01)	0.510 (0.664)	0.918 (0.930)	0.995 (0.996)
	(1.2, 0.02)	0.019 (0.341)	0.534 (0.644)	0.844 (0.866)
	(2.3, 0.04)	0.000 (0.155)	0.010 (0.324)	0.315 (0.489)
(150, 150)	(0.7, 0.01)	0.773 (0.838)	0.987 (0.987)	0.999 (0.999)
	(1.2, 0.02)	0.228 (0.479)	0.768 (0.801)	0.959 (0.961)
	(2.3, 0.04)	0.000 (0.203)	0.175 (0.378)	0.588 (0.664)
(250, 250)	(0.7, 0.01)	0.956 (0.962)	0.999 (0.999)	1.000 (1.000)
	(1.2, 0.02)	0.557 (0.663)	0.936 (0.938)	0.990 (0.990)
	(2.3, 0.04)	0.012 (0.267)	0.507 (0.575)	0.828 (0.836)

**Table 3:** Simulated power of the non-inferiority test (15) (numbers in brackets) and the equivalence test (16) for the NPH scenario (20) with $\theta _2=(2,3.4)$ at four different time points $t_0=0.2,0.6,2.7,3.2$ for different sample sizes and equivalence margins $\delta$. The nominal level is chosen as $\alpha =0.05$

$(n_1,n_2)$	$(t_0,S_1(t_0)-S_2(t_0))$	$\delta =0.1$	$\delta =0.15$	$\delta =0.2$
(20, 20)	(0.2, 0.01)	0.964 (0.964)	0.996 (0.999)	1.000 (1.000)
	(0.6, 0.04)	0.372 (0.448)	0.712 (0.712)	0.891 (0.893)
	(2.7, 0.04)	0.000 (0.124)	0.000 (0.219)	0.000 (0.297)
	(3.2, 0.01)	0.000 (0.179)	0.000 (0.284)	0.000 (0.374)
(50, 50)	(0.2, 0.01)	1.000 (1.000)	1.000 (1.000)	1.000 (1.000)
	(0.6, 0.04)	0.637 (0.640)	0.938 (0.938)	0.997 (0.997)
	(2.7, 0.04)	0.000 (0.151)	0.047 (0.339)	0.436 (0.563)
	(3.2, 0.01)	0.000 (0.258)	0.054 (0.468)	0.481 (0.684)
(100, 100)	(0.2, 0.01)	1.000 (1.000)	1.000 (1.000)	1.000 (1.000)
	(0.6, 0.04)	0.818 (0.841)	0.995 (0.995)	1.000 (1.000)
	(2.7, 0.04)	0.001 (0.246)	0.442 (0.555)	0.803 (0.833)
	(3.2, 0.01)	0.006 (0.406)	0.558 (0.728)	0.869 (0.921)
(150, 150)	(0.2, 0.01)	1.000 (1.000)	1.000 (1.000)	1.000 (1.000)
	(0.6, 0.04)	0.927 (0.927)	1.000 (1.000)	1.000 (1.000)
	(2.7, 0.04)	0.206 (0.326)	0.678 (0.701)	0.922 (0.928)
	(3.2, 0.01)	0.267 (0.553)	0.797 (0.859)	0.903 (0.984)
(250, 250)	(0.2, 0.01)	1.000 (1.000)	1.000 (1.000)	1.000 (1.000)
	(0.6, 0.04)	0.987 (0.987)	1.000 (1.000)	1.000 (1.000)
	(2.7, 0.04)	0.437 (0.471)	0.882 (0.883)	0.993 (0.993)
	(3.2, 0.01)	0.609 (0.742)	0.968(0.975)	0.998 (0.998)

**Table 4:** Simulated power of the non-parametric approach for the non-inferiority test (numbers in brackets) and the equivalence test for the NPH scenario (20) with $\theta _2=(2,3.4)$ at four different time points for different sample sizes and equivalence margins. The nominal level is chosen as $\alpha =0.05$

$(n_1,n_2)$	$(t_0,S_1(t_0)-S_2(t_0))$	$\delta =0.1$	$\delta =0.15$	$\delta =0.2$
(20, 20)	(0.2, 0.01)	0.709 (0.755)	0.951 (0.953)	0.968 (0.968)
	(0.6, 0.04)	0.144 (0.289)	0.529 (0.606)	0.651 (0.706)
	(2.7, 0.04)	0.000 (0.124)	0.000 (0.168)	0.000 (0.263)
	(3.2, 0.01)	0.000 (0.169)	0.000 (0.229)	0.000 (0.298)
(50, 50)	(0.2, 0.01)	0.961 (0.963)	0.999 (0.999)	1.000 (1.000)
	(0.6, 0.04)	0.389 (0.447)	0.774 (0.779)	0.949 (0.949)
	(2.7, 0.04)	0.000 (0.150)	0.000 (0.272)	0.130 (0.425)
	(3.2, 0.01)	0.000 (0.230)	0.000 (0.349)	0.125 (0.506)
(100, 100)	(0.2, 0.01)	0.998 (0.998)	1.000 (1.000)	1.000 (1.000)
	(0.6, 0.04)	0.598 (0.600)	0.946 (0.946)	0.998 (0.998)
	(2.7, 0.04)	0.000 (0.188)	0.183 (0.425)	0.590 (0.657)
	(3.2, 0.01)	0.000 (0.307)	0.232 (0.581)	0.668 (0.807)
(150, 150)	(0.2, 0.01)	1.000 (1.000)	1.000 (1.000)	1.000 (1.000)
	(0.6, 0.04)	0.772 (0.772)	0.991 (0.991)	1.000 (1.000)
	(2.7, 0.04)	0.000 (0.255)	0.465 (0.530)	0.790 (0.806)
	(3.2, 0.01)	0.000 (0.411)	0.537 (0.702)	0.857 (0.902)
(250, 250)	(0.2, 0.01)	1.000 (1.000)	1.000 (1.000)	1.000 (1.000)
	(0.6, 0.04)	0.906 (0.906)	0.999 (0.999)	1.000 (1.000)
	(2.7, 0.04)	0.229 (0.329)	0.703 (0.718)	0.949 (0.949)
	(3.2, 0.01)	0.316 (0.575)	0.848 (0.889)	0.980 (0.984)

## Case study

**Fig. 6:**
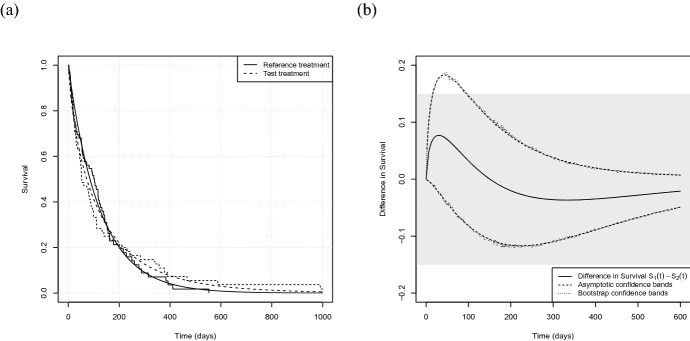
**a** Survival curves (Kaplan–Meier curves and Weibull models) for the veteran data. Solid lines correspond to the reference group, dashed lines to the test treatment. **b** Difference in survival, pointwise confidence bands obtained by the asymptotic approach (dashed) and bootstrap (dotted), respectively, on the interval $\left[ 0,600\right]$. The shaded area indicates the equivalence margins with $\delta =0.15$

In the following we will investigate a well known benchmark dataset regarding survival analysis. The data set veteran from Veteran’s Administration Lung Cancer Trial (Kalbfleisch and Prentice 2011), implemented in the R package survival (Therneau 2020), describes a two-treatment, randomized trial for lung cancer. In this trial, male patients with advanced inoperable lung cancer were allocated to either a standard therapy (reference treatment, $\ell =1$) or a chemotherapy (test treatment, $\ell =2$). Numerous covariates were documented, including time to death for each patient, which is the primary endpoint of our analysis. In total 137 observations, allocated to $n_1=69$ patients in the reference group and $n_2=68$ in the test group, are given. The code reproducing the results presented in the following has been implemented in the R package EquiSurv (Möllenhoff 2020). As our analysis is model-based, we start with a model selection step. More precisely we split the data into the reference group and the test group and assume six different distributions, that is a Weibull distribution, an exponential distribution, a Gaussian distribution, a logistic distribution, a log-normal distribution and a log-logistic distribution, respectively. We fit the corresponding models separately per treatment group, resulting in 12 models in total. Finally we compare for each group the six different models using Akaike’s Information Criterion, AIC (Sakamoto et al. 1986). It turns out that for the group receiving the reference treatment the Weibull and the exponential model provide the best fits (AICs are given by 749.1, 747.1, 799.9, 794.7, 755.1 and 758.1 in the order of the models mentioned above) whereas in the test group the log-logistic, the log-normal and the Weibull model are the best ones (AICs given by 749.1, 750.1 and 751.7, respectively). Therefore we decide to base our analyses on Weibull models for both groups. However, note that all tests could also be performed assuming different distributions for each treatment. We assume the censoring times to be exponentially distributed and maximizing the likelihood (1) yields $\hat{\theta }_1=(4.82,1.01),\ \hat{\psi }_1=0.00063$ and $\hat{\theta }_2=(4.76,1.3),\ \hat{\psi }_2=0.00046.$ These low censoring rates can be explained by the fact that in total only 9 of the 137 individuals have been censored, precisely 7.3% in the reference treatment group and 5.8% in the test treatment group, respectively. Fig. 6a displays the corresponding Weibull models and the non-parametric analogue given by Kaplan–Meier curves. It turns out that for both treatment groups the parametric and the non-parametric curves are very close to each other. Further we observe that the survival curves of the two treatment groups cross each other which indicates that the assumption of proportional hazards is not justified here. Indeed, the hazard ratio ranges from 0.55 to 1.93 from the first time of event (3 days) until the end of the observational period (999 days) and therefore an analysis using a proportional hazards model is actually not applicable here. The p-value of the log-rank test is 0.928 and thus does not detect any difference between the two groups.

We will now perform a similar analysis using the parametric models and the theory derived in Sect. 2. For the sake of brevity we will only consider difference in survival, analyses concerning the (log) hazard ratio can be conducted in the same manner. We consider the first 600 days of the trial. We set $\alpha =0.05$ and calculate lower and upper $(1-\alpha )$-pointwise confidence bands according to (5) at several points $0\le t \le 600$. Estimates of the variance $\hat{\sigma }_{\Delta }$ are obtained by both, the asymptotic approach and bootstrap as described in Algorithm 1, respectively. Figure 6b displays the estimated difference curve $\Delta (t,\hat{\theta }_1,\hat{\theta }_2)=S_1(t,\hat{\theta }_1)-S_2(t,\hat{\theta }_2)$ and the pointwise confidence bands on the interval $\left[ 0,600\right]$. We note that there is almost no difference between the two methods, meaning that the asymptotic and the bootstrap approach yield very similar results here, which can be explained by the rather high sample size combined with the very low rate of censoring. We start our analysis considering $t_0=80$, which is close to the median survival of both treatment groups. The difference in survival is $\Delta (80,\hat{\theta }_1,\hat{\theta }_2)=0.047$ and the asymptotic confidence interval at this point is given by $\left[ -0.068,0.163\right]$, while the bootstrap yields $\left[ -0.067, 0.162\right]$. Note that these are two-sided $90\%$-confidence intervals, as we use $95\%$-upper and $95\%$-lower confidence bands for the test decisions. Of note, if we assume an alternative censoring distribution, namely a uniform distribution, we obtain $\left[ -0.076, 0.175\right]$, which is a bit wider but still very close to the other two intervals. Continuing with the narrower confidence bands and investigating the hypotheses (11) and (12) we observe that both, non-inferiority and equivalence can be claimed for all $\delta >0.163$ (0.162), respectively. Consequently, for $\delta =0.15$, which is indicated by the shaded area in Fig. 6b, $H_0$ cannot be rejected in both cases, meaning in particular that the treatments cannot be considered equivalent with respect to the 80-days survival. Figure 6b further displays these investigations for $\delta =0.15$ simultaneously at all time points under consideration. We conclude that the chemotherapy is non-inferior to the standard therapy regarding survival after 96 days, as this is the earliest time point where the upper confidence bound is smaller than $\delta =0.15$, which means that the null hypothesis can be rejected for the first time. The same conclusion can be made concerning equivalence as for all t in $\left[ 0,600\right]$, the lower confidence bands are completely contained in the rejection region, meaning that they are larger than $-\delta =-0.15$. Consequently we can conclude that both treatments are equivalent concerning for example the 6-months or one-year-survival, respectively. Finally we further observe that considering for instance $\delta =0.2$, equivalence would be claimed at all time points under consideration.

## Discussion

In this paper, we addressed the problem of survival analysis in the presence of non-proportional hazards. Here, commonly used methods, as Cox’s proportional hazards model or the log-rank test, are not optimal and suffer from a loss of power. Therefore we proposed another approach for investigating equivalence or non-inferiority of time-to-event outcomes based on the construction of (pointwise) confidence bands and applicable irrespectively of the shape of the hazard ratio. We proposed two ways of constructing confidence bands for both the (log) hazard ratio and the difference of the survival curves. One is based on asymptotic investigations, and the other on a parametric bootstrap procedure. Both approaches show a similar performance. The latter has the advantage that it can be used for small sample sizes and does not require calculating the asymptotic variance. Our approach provides a framework for investigating survival in many ways. Apart from specific time points entire periods of time can be observed and the difference in survival compared to a specific equivalence margin. We demonstrated that the presence of non-proportional hazards does not affect the performance of the confidence bands and the non-inferiority and equivalence tests, respectively, which means that they do not rely on this assumption. Consequently, this framework provides a much more flexible tool than standard methodology.

Our methods are based on estimating parametric survival functions, which can be an effective tool once the model’s suitability is proven. The latter has to be assessed in a preliminary study, using, for instance, model selection criteria as the AIC. If a model has to be prespecified, which can be necessary in some clinical trials, the model-selection step cannot be performed properly and the model may suffer from misspecification. To this end we investigated the robustness of our approach and it turned out that if the underlying distribution of the event times is not correctly specified a type I error inflation occurred in some configurations with a relatively large equivalence/non-inferiority margin. In general, the choice of the margin should clearly be determined by clinicians and practical considerations rather than by statistical properties. If one wants to reduce the risk of a type I error, a conservative, i.e. smaller, margin should be chosen, as no type I error inflation occurred for these configurations. However, we note that for those margins the power, that is correctly claiming equivalence/non-inferiority, can be decreased. Further, as another non-parametric alternative one could also investigate confidence bands based on the classical bootstrap for survival data (Efron 1981; Akritas 1986).

In clinical research there might be situations where it makes sense to consider different metrics as the (maximum) difference between survival curves. Such a metric could, for example, be the area between the survival curves, that is the difference in mean survival times. We leave the investigation and extension of the proposed methods to this situation for future research.

### Supplementary Information

Below is the link to the electronic supplementary material.

Supplementary file 1 (pdf 192 KB)

## Appendix

### A1. Proof of Lemma 1

In the following we prove that the equivalence test described in Eq. (16) is an asymptotic $\alpha$-level test. Under the null hypothesis in Eq. (12) the probability of rejecting $H_0$ is given by

22 $$\begin{aligned}&\lim _{n_1,n_2\rightarrow \infty }{\mathbb {P}}_{H_0}(U_\Delta (t_0,\hat{\theta }_1,\hat{\theta }_2)\le \delta , L_\Delta (t_0,\hat{\theta }_1,\hat{\theta }_2)\ge -\delta )\nonumber \\&\quad =\lim _{n_1,n_2\rightarrow \infty }{\mathbb {P}}_{H_0}(-\delta -\Delta (t_0,\theta _1,\theta _2)+z_{1-\alpha } \hat{\sigma }_\Delta \le \Delta (t_0,\hat{\theta }_1,\hat{\theta }_2)-\Delta (t_0,\theta _1,\theta _2)\nonumber \\&\quad \le \delta -\Delta (t_0,\theta _1,\theta _2)- z_{1-\alpha } \hat{\sigma }_\Delta ). \end{aligned}$$

We now consider the margin of the null hypothesis (that is $\Delta (t_0,\theta _1,\theta _2)=\pm \delta$). Without loss of generality we assume that $\Delta (t_0,\theta _1,\theta _2)=\delta$ and (22) becomes

$$\begin{aligned}&\lim _{n_1,n_2\rightarrow \infty }{\mathbb {P}}(-2\delta +z_{1-\alpha } \hat{\sigma }_\Delta \le \Delta (t_0,\hat{\theta }_1,\hat{\theta }_2)-\Delta (t_0,\theta _1,\theta _2)\le - z_{1-\alpha } \hat{\sigma }_\Delta ) \\&\quad \le \lim _{n_1,n_2\rightarrow \infty }{\mathbb {P}}(\Delta (t_0,\hat{\theta }_1,\hat{\theta }_2)-\Delta (t_0,\theta _1,\theta _2)\le - z_{1-\alpha } \hat{\sigma }_\Delta ) \\&\quad =\lim _{n_1,n_2\rightarrow \infty }{\mathbb {P}}(U_\Delta (t_0,\hat{\theta }_1,\hat{\theta }_2)\le \Delta (t_0,\theta _1,\theta _2))\\&\quad \le \ \ \alpha , \end{aligned}$$

due to (6). The same arguments hold for $\Delta (t_0,\theta _1,\theta _2)=-\delta$. When considering the interior of the null hypothesis, that is $| \Delta (t_0,\theta _1,\theta _2)|>\delta$, the probability in Eq. (22) gets even smaller and, moreover, converges to zero due to the asymptotic normality of $\sqrt{n}(\Delta (t_0,\hat{\theta }_1,\hat{\theta }_2)-\Delta (t_0,\theta _1,\theta _2))$, as $\delta -\Delta (t_0,\theta _1,\theta _2)<0$ or $-\delta -\Delta (t_0,\theta _1,\theta _2)>0$.

### A2. Asymptotic variances for a Weibull distribution

In order to calculate confidence bands as given in Eq. (5) and (8), respectively, we need a formula for the gradients yielding asymptotic variances as described in Eq. (4) and (7). We now present an exemplary calculation assuming a Weibull distribution, that is $F_\ell (t,\theta _\ell )=1-\exp {\left\{ -(t/\theta _{\ell ,2})^{\theta _{\ell ,1}}\right\} }$, $\ell =1,2$. The gradients are given by

$$\begin{aligned} \tfrac{\partial }{\partial \theta _\ell }S_\ell (t,\theta _\ell )= \exp {\left\{ -(t/\theta _{\ell ,2})^{\theta _{\ell ,1}}\right\} }\cdot \big (-(t/\theta _{\ell ,2})^{\theta _{\ell ,2}}\cdot \log (t/\theta _{\ell ,2}),t^{\theta _{\ell ,1}}\cdot \theta _{\ell ,1}\cdot \theta _{\ell ,2}^{-\theta _{\ell ,1}-1}\big ), \end{aligned}$$

$$ \ell =1,2,$$ and

$$\begin{aligned} \tfrac{\partial }{\partial \theta _\ell }\log {h_\ell (t,\theta _\ell )}=\big (1/\theta _{\ell ,1}+\log {(t/\theta _{\ell ,2})}, -\theta _{\ell ,1}/\theta _{\ell ,2} \big ),\ \ell =1,2. \end{aligned}$$

Substituting these gradients in the formula (4) and (7), respectively and using the MLE and the corresponding Fisher information matrix obtained via maximizing (1) finally yields the asymptotic variance of $\Delta (t,\theta _1,\theta _2)$ and $r(t,\theta _1,\theta _2)$, respectively. The estimates can be obtained by using standard statistical software as for example R, the concrete implementation can be found in the R package EquiSurv (Möllenhoff 2020).
