# Supplementary material for: Investigating non-inferiority or equivalence in time-to-event data under non-proportional hazards
Source: Lifetime Data Anal. 2023 Jan 28;29(3):483–507. doi: 10.1007/s10985-023-09589-5 (PMC10258187; doi:10.1007/s10985-023-09589-5)
Supplement: Supplementary file 1 — (pdf 192 KB) [file 10985_2023_9589_MOESM1_ESM.pdf]

Supplementary Material to: Investigating non-inferiority or equivalence in time-to-event data under non-proportional hazards

July 5, 2022

In the following we will present numerous additional simulation results concerning the robustness of the methods (see Section 1), the effect of the censoring rate (see Section 2), additional results concerning the type I error of the test (Section 3) and the results for comparing survival over an entire period of time (Section 4).

1 Robustness

1.1 Coverage probabilities in case of a misspecified censoring distribution

In the following we investigate the effect of misspecifying the censoring distribution by considering the NPH scenario (20). Of note, this misspecification does only affect the bootstrap bands and not the asymptotic confidence bands, as those do not take the censoring mechanism into account. Therefore, we assumed a uniform distribution of the censoring times instead of the true underlying exponential distribution. Figure 1 displays the coverage probabilities compared to the correctly specified situation. It turns out that the effect of misspecification is rather small. The bands tend to be slightly more conservative as the coverage probabilities are close to 1 for the small sample size setting of $n_1 = n_2 = 20$. However, in general they are above the desired 0.95 in all scenarios and for increasing sample sizes this approximation is actually very close. This holds for both, the bands for $S_1 - S_2$ and the bands for the log hazard ratio. We conclude that the bands are very robust against missspecification of the censoring distribution.

1.2 Type I errors in case of missspecification

In order to investigate Type I errors in presence of misspecification we generate survival times according to a log-logistic distribution, but assume the data to be Weibull distributed. Further

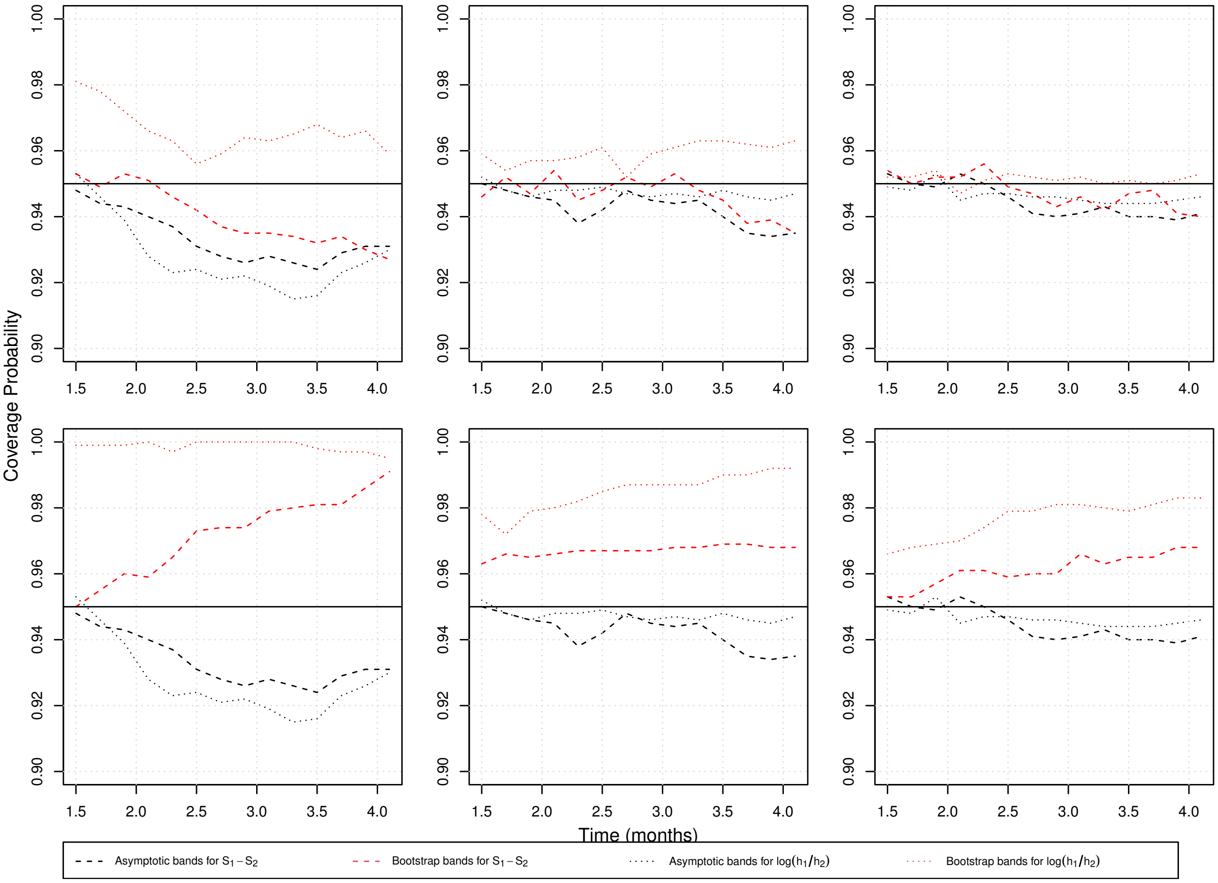

Figure 1: Simulated coverage probabilities for the NPH scenario (20) with correctly specified censoring distribution (first row) and a missspecified censoring distribution (second row) at different time points for sample sizes of $n_1 = n_2 = 20,\ 50,\ 100$ (left, middle, right column). The dashed lines correspond to the confidence bands for the difference of the survival functions, the dotted lines to the confidence bands for the log hazard ratio. Black lines display the asymptotic bands, red lines the confidence bands based on bootstrap, respectively.

we assume the censoring times to be uniformly distributed on an interval $[0, c_\ell]$, where c_ℓ is again chosen such that a censoring rate of approximately 20% results, $\ell = 1, 2$. We now choose a time range (in months) given by $\mathcal{T} = [0, 12]$, such that the latest point of follow up is after 12 months and as described in scenario (21) of the main manuscript the set of parameters is given by $\theta_1 = (1.5, 2.6),\ \theta_2 = (2.1, 3.9)$, where θ_1 corresponds to the reference model, see Figure 1(c) of the main manuscript.

Table 1 displays the Type I errors of the non-inferiority test (15) (numbers in brackets) and the equivalence test (16). It becomes obvious that when considering the margin of the null hypotheses in (11) and (12), respectively, there is a type I error inflation for the two larger margins, that is $\delta = 0.15, 0.2$. This inflation even increases with growing sample sizes and occurs for both tests, the non-inferiority test (15) and the equivalence test (16), respectively. For all other configurations the type I error is well below the nominal level of 0.05 and hence we conclude that the robustness of the proposed methods strongly depends on the chosen scenario and the time point under consideration. However, type I error inflation can be reduced if a rather moderate non-inferiority/equivalence margin δ is chosen.

Table 2 displays the corresponding simulated Type I errors for the non-parametric approach. The approximation of the level is very precise for sufficiently large sample sizes whereas an Type I error inflation of the non-inferiority test becomes visible for smaller margins of $\delta = 0.1, 0.15$ and small sample sizes, that is $n_1 = n_2 = 20$. However, as the non-parametric approach doesn't rely on any assumption of the distribution, this effect cannot be explained by misspecification but by a general reduced accuracy due to a small sample size and another underlying distribution (log-logistic). In general we conclude that in case of misspecified models, depending on the equivalence margin, the tests may exceed their nominal level. Consequently the model selection step should be done very carefully. However, if the margin is rather conservative, the procedure turns out to be reasonably robust.

2 The effect of the censoring rate

For the following analysis we consider the NPH Scenario (20) with a fixed sample size of $(n_1, n_2) = (100, 100)$ in order to investigate the effect of different censoring rates on the procedure. Therefore we vary the parameters $\psi_\ell,\ \ell = 1, 2$, such that given the latest time point of follow-up by $t_{max} = 9$, between 10% and 75% of the individuals are censored. Precisely, we also consider unbalanced situations where censoring rates are different across the two groups. Again, the results are compared to the non-parametric approach described in Com-Nougue et al. (1993). For the type I error, the censoring parameters $\psi_\ell,\ \ell = 1, 2$, range from 0.035 up to 0.78, resulting in a proportion of 10% up to 85% of the individuals being censored. Details on that are summarized in Table 3.

The results for the type I errors are presented in Table 4 and 5. It turns out that both, the

Table 1: Simulated type I errors of the parametric non-inferiority test (15) (numbers in brackets) and the equivalence test (16) for the log-logistic scenario of misspecification (21) at different time points for different sample sizes and equivalence margins. The significance level is chosen as $\alpha = 0.05$. Scenarios not falling under the null hypothesis are marked with " $-$ ".

(n_1, n_2)	$(t_0, S_2(t_0) - S_1(t_0))$	$\delta = 0.1$	$\delta = 0.15$	$\delta = 0.2$
	$(0.7, 0.1)$	0.033 (0.071)	-	-
$(20, 20)$	$(1.1, 0.15)$	0.004 (0.027)	0.037 (0.062)	-
	$(1.8, 0.2)$	0.000 (0.011)	0.000 (0.021)	0.034 (0.072)
	$(0.7, 0.1)$	0.037 (0.038)	-	-
$(50, 50)$	$(1.1, 0.15)$	0.006 (0.009)	0.089 (0.090)	-
	$(1.8, 0.2)$	0.000 (0.000)	0.022 (0.023)	0.091 (0.091)
	$(0.7, 0.1)$	0.042 (0.044)	-	-
$(100, 100)$	$(1.1, 0.15)$	0.001 (0.001)	0.090 (0.102)	-
	$(1.8, 0.2)$	0.000 (0.000)	0.012 (0.012)	0.154 (0.157)
	$(0.7, 0.1)$	0.035 (0.046)	-	-
$(150, 150)$	$(1.1, 0.15)$	0.001 (0.001)	0.123 (0.123)	-
	$(1.8, 0.2)$	0.000 (0.000)	0.009 (0.009)	0.206 (0.214)

new approach and the non-parametric approach, control their level in all configurations. As expected, for increasing censoring rates, the approximation of the level gets less precise and both equivalence tests rather conservative. This effect particularly occurs for small equivalence/non-inferiority margins and is stronger for the non-parametric approach. However, for the non-inferiority test, the approximation of the level remains very accurate with both methods, even if 75% of the individuals are censored.

Tables 6 and 7 display the power of the parametric and the non-parametric test, respectively. As expected, the power generally decreases with increasing censoring rates, which becomes particularly visible at the later time points 2.7 and 3.2. Further it becomes obvious that the parametric test achieves a higher power in all scenarios under consideration. The parametric test still achieves reasonable results for high censoring rates if the threshold is not too small. To give an example, in the case of censoring rates of 75% and a threshold of $\delta = 0.2$, the power of the parametric non-inferiority test still lies between 0.486 and 1.000, whereas the non-parametric version attains values between 0.355 and 1.000, respectively.

Table 2: Simulated type I errors of the non-parametric approach for the non-inferiority test (numbers in brackets) and the equivalence test in the log-logistic scenario of misspecification (21) at different time points for different sample sizes and equivalence margins. The significance level is chosen as $\alpha = 0.05$. Scenarios not falling under the null hypothesis are marked with " − ".

(n_1, n_2)	$(t_0, S_2(t_0) - S_1(t_0))$	$\delta = 0.1$	$\delta = 0.15$	$\delta = 0.2$
	$(0.7, 0.1)$	0.045 (0.097)	-	-
$(20, 20)$	$(1.1, 0.15)$	0.004 (0.031)	0.029 (0.075)	-
	$(1.8, 0.2)$	0.000 (0.009)	0.000 (0.036)	0.007 (0.056)
	$(0.7, 0.1)$	0.049 (0.062)	-	-
$(50, 50)$	$(1.1, 0.15)$	0.004 (0.017)	0.064 (0.066)	-
	$(1.8, 0.2)$	0.000 (0.000)	0.001 (0.010)	0.063 (0.066)
	$(0.7, 0.1)$	0.065 (0.065)	-	-
$(100, 100)$	$(1.1, 0.15)$	0.003 (0.003)	0.047 (0.047)	-
	$(1.8, 0.2)$	0.000 (0.000)	0.007 (0.007)	0.054 (0.054)
	$(0.7, 0.1)$	0.060 (0.060)	-	-
$(150, 150)$	$(1.1, 0.15)$	0.004 (0.004)	0.043 (0.043)	-
	$(1.8, 0.2)$	0.001 (0.001)	0.003 (0.003)	0.049 (0.049)

Table 3: Configurations of the censoring parameters for the two groups in the NPH scenario (20).

	H_0		H_1	
Censoring rates	ψ_1	ψ_1^1	ψ_1	ψ_2^1
(10%, 10%)	0.035	0.05	0.035	0.035
(25%, 25%)	0.1	0.14	0.1	0.1
(25%, 50%)	0.1	0.35	0.1	0.25
(50%, 50%)	0.27	0.35	0.27	0.25
(75%, 50%)	0.65	0.35	0.65	0.25
(75%, 75%)	0.65	0.78	0.65	0.58

3 Additional tables for the type I errors

In this section all tables which were summarized and analysed in the main paper are presented. Tables 8, 9, 10 and 11 display the exact type I errors for both procedures and both scenarios and all chosen configurations.

Table 4: Simulated type I errors of the parametric non-inferiority test (numbers in brackets) and the equivalence test for the NPH scenario (20) with $n_1 = n_2 = 100$ at different time points for different censoring rates and equivalence margins. The significance level is chosen as $\alpha = 0.05$. Scenarios not falling under the null hypothesis are marked with " $-$ ".

Censoring rates	$(t_0, S_1(t_0) - S_2(t_0))$	$\delta = 0.1$	$\delta = 0.15$	$\delta = 0.2$
$(10\%, 10\%)$	$(1.9, 0.1)$	**0.017 (0.057)**	-	-
	$(2.4, 0.15)$	0.002 (0.010)	**0.047 (0.048)**	-
	$(3, 0.2)$	0.001 (0.001)	0.005 (0.006)	**0.043 (0.043)**
$(25\%, 25\%)$	$(1.9, 0.1)$	**0.002 (0.057)**	-	-
	$(2.4, 0.15)$	0.000 (0.008)	**0.055 (0.055)**	-
	$(3, 0.2)$	0.000 (0.001)	0.006 (0.006)	**0.038 (0.038)**
$(25\%, 50\%)$	$(1.9, 0.1)$	**0.000 (0.056)**	-	-
	$(2.4, 0.15)$	0.000 (0.003)	**0.041 (0.044)**	-
	$(3, 0.2)$	0.000 (0.000)	0.009 (0.009)	**0.049 (0.049)**
$(50\%, 50\%)$	$(1.9, 0.1)$	**0.000 (0.052)**	-	-
	$(2.4, 0.15)$	0.000 (0.005)	**0.044 (0.047)**	-
	$(3, 0.2)$	0.000 (0.001)	0.007 (0.009)	**0.057 (0.057)**
$(75\%, 50\%)$	$(1.9, 0.1)$	**0.000 (0.053)**	-	-
	$(2.4, 0.15)$	0.000 (0.009)	**0.020 (0.051)**	-
	$(3, 0.2)$	0.000 (0.001)	0.002 (0.017)	**0.062 (0.069)**
$(75\%, 75\%)$	$(1.9, 0.1)$	**0.000 (0.041)**	-	-
	$(2.4, 0.15)$	0.000 (0.013)	**0.000 (0.052)**	-
	$(3, 0.2)$	0.000 (0.005)	0.000 (0.015)	**0.013 (0.045)**

4 Comparing survival over an entire interval

We now consider the entire observational period (that is $\mathcal{T} = [0, 9]$) and reject the null hypothesis in (16) according to the test defined in Section 2.2.2 of the main paper. For both scenarios under the null hypothesis, that is the PH sceneario and the NPH sceneario, respectively, the maximum distance between the survival curves, that is $\max_{t \in [0,9]} |S_1(t, \theta_1) - S_2(t, \theta_2)|$, is 0.2. Hence for all three different equivalence margins the situation reflects the null hypothesis and moreover, for a choice of $\delta = 0.2$ the margin of H_0. Table 12 shows the type I errors. For both scenarios we observe that the test keeps its nominal level for all configurations. For very small samples, namely $n_1 = n_2 = 20$ the equivalence test turns out to be very conservative, whereas on the margin of the null hypothesis the non-inferiority test still attains a reasonable type I error of 0.03 for the NPH scenario and 0.042 for the PH scenario, respectively. In general, for

Table 5: Simulated type I errors of the non-parametric non-inferiority test (numbers in brackets) and the equivalence test for the NPH scenario (20) with $n_1 = n_2 = 100$ at different time points for different censoring rates and equivalence margins. The significance level is chosen as $\alpha = 0.05$. Scenarios not falling under the null hypothesis are marked with " − ".

Censoring rates	$(t_0, S_1(t_0) - S_2(t_0))$	$\delta = 0.1$	$\delta = 0.15$	$\delta = 0.2$
$(10\%, 10\%)$	$(1.9, 0.1)$	**0.000 (0.056)**	-	-
	$(2.4, 0.15)$	0.000 (0.011)	**0.042 (0.044)**	-
	$(3, 0.2)$	0.000 (0.002)	0.008 (0.008)	**0.049 (0.049)**
$(25\%, 25\%)$	$(1.6, 0.1)$	**0.012 (0.037)**	-	-
	$(2.3, 0.15)$	0.000 (0.005)	**0.047 (0.051)**	-
	$(4, 0.2)$	0.000 (0.001)	0.009 (0.014)	**0.044 (0.044)**
$(25\%, 50\%)$	$(1.9, 0.1)$	**0.000 (0.055)**	-	-
	$(2.4, 0.15)$	0.000 (0.012)	**0.014 (0.037)**	-
	$(3, 0.2)$	0.000 (0.002)	0.000 (0.005)	**0.046 (0.047)**
$(50\%, 50\%)$	$(1.9, 0.1)$	**0.000 (0.054)**	-	-
	$(2.4, 0.15)$	0.000 (0.014)	**0.000 (0.043)**	-
	$(3, 0.2)$	0.000 (0.002)	0.002 (0.012)	**0.044 (0.049)**
$(75\%, 50\%)$	$(1.9, 0.1)$	**0.000 (0.062)**	-	-
	$(2.4, 0.15)$	0.000 (0.023)	**0.000 (0.040)**	-
	$(3, 0.2)$	0.015 (0.006)	0.002 (0.028)	**0.015 (0.053)**
$(75\%, 75\%)$	$(1.9, 0.1)$	**0.000 (0.063)**	-	-
	$(2.4, 0.15)$	0.000 (0.022)	**0.000 (0.055)**	-
	$(3, 0.2)$	0.000 (0.021)	0.000 (0.029)	**0.000 (0.062)**

both scenarios the type I errors approach zero in the interior of the null hypothesis and 0.05 on the margin.

4.0.1 Power for comparing survival over an entire interval

We now consider again the entire observational period and the PH scenario (19) with $\theta_2 = (1.5, 3.7)$ and the NPH scenario (20) with $\theta_2 = (2, 3.4)$. For the first scenario we have $\max_{t\in[0,9]} S_1(t,\theta_1) - S_2(t,\theta_2) = 0.05$, for the latter the maximum distance between the survival curves is given by 0.07. Further we observe a third scenario, where we choose identical survival curves, that is $\theta_2 = \theta_1$, resulting in a maximum absolute difference of

$$\max_{t\in[0,9]} |S_1(t,\theta_1) - S_2(t,\theta_2)| = 0.$$

Table 6: Simulated power of the parametric non-inferiority test (numbers in brackets) and the equivalence test for the NPH scenario (20) with $n_1 = n_2 = 100$ at different time points for different censoring rates and equivalence margins. The significance level is chosen as $\alpha = 0.05$.

Censoring rates	$(t_0, S_1(t_0) - S_2(t_0))$	$\delta = 0.1$	$\delta = 0.15$	$\delta = 0.2$
$(10\%, 10\%)$	$(0.2, 0.01)$	1.000 (1.000)	1.000 (1.000)	1.000 (1.000)
	$(0.6, 0.04)$	0.862 (0.862)	0.998 (0.998)	1.000 (1.000)
	$(2.7, 0.04)$	0.025 (0.265)	0.528 (0.595)	0.855 (0.862)
	$(3.2, 0.01)$	0.064 (0.449)	0.646 (0.790)	0.936 (0.956)
$(25\%, 25\%)$	$(0.2, 0.01)$	1.000 (1.000)	1.000 (1.000)	1.000 (1.000)
	$(0.6, 0.04)$	0.818 (0.841)	0.995 (0.995)	1.000 (1.000)
	$(2.7, 0.04)$	0.001 (0.246)	0.442 (0.555)	0.803 (0.833)
	$(3.2, 0.01)$	0.006 (0.406)	0.558 (0.728)	0.869 (0.921)
$(25\%, 50\%)$	$(0.2, 0.01)$	1.000 (1.000)	1.000 (1.000)	1.000 (1.000)
	$(0.6, 0.04)$	0.825 (0.825)	0.996 (0.996)	1.000 (1.000)
	$(2.7, 0.04)$	0.000 (0.228)	0.401 (0.513)	0.751 (0.778)
	$(3.2, 0.01)$	0.000 (0.379)	0.413 (0.662)	0.808 (0.873)
$(50\%, 50\%)$	$(0.2, 0.01)$	1.000 (1.000)	1.000 (1.000)	1.000 (1.000)
	$(0.6, 0.04)$	0.785 (0.785)	0.994 (0.994)	0.999 (0.999)
	$(2.7, 0.04)$	0.000 (0.209)	0.344 (0.490)	0.683 (0.722)
	$(3.2, 0.01)$	0.000 (0.345)	0.314 (0.626)	0.712 (0.827)
$(75\%, 50\%)$	$(0.2, 0.01)$	1.000 (1.000)	1.000 (1.000)	1.000 (1.000)
	$(0.6, 0.04)$	0.728 (0.728)	0.984 (0.984)	0.999 (0.999)
	$(2.7, 0.04)$	0.000 (0.158)	0.065 (0.347)	0.398 (0.537)
	$(3.2, 0.01)$	0.000 (0.225)	0.010 (0.415)	0.319 (0.600)
$(75\%, 75\%)$	$(0.2, 0.01)$	1.000 (1.000)	1.000 (1.000)	1.000 (1.000)
	$(0.6, 0.04)$	0.691 (0.691)	0.981 (0.981)	0.999 (0.999)
	$(2.7, 0.04)$	0.000 (0.138)	0.000 (0.289)	0.258 (0.486)
	$(3.2, 0.01)$	0.000 (0.205)	0.000 (0.348)	0.120 (0.525)

We further add another sample size, that is $n_1 = n_2 = 200$. Table 13 displays the power of the test. The results are in line with the findings for the pointwise version. For both tests the power increases with increasing sample sizes and larger non-inferiority/equivalence margins. For sufficiently large sample sizes we observe a reasonable power for both tests. For instance, considering a sample size of $n_1 = n_2 = 100$ the maximum power is given by 0.852 for the equivalence test and by 0.929 for the non-inferiority test, respectively.

Table 7: Simulated power of the non-parametric non-inferiority test (numbers in brackets) and the equivalence test for the NPH scenario (20) with $n_1 = n_2 = 100$ at different time points for different censoring rates and equivalence margins. The significance level is chosen as $\alpha = 0.05$.

Censoring rates	$(t_0, S_1(t_0) - S_2(t_0))$	$\delta = 0.1$	$\delta = 0.15$	$\delta = 0.2$
(10%, 10%)	(0.2, 0.01)	0.999 (0.999)	1.000 (1.000)	1.000 (1.000)
	(0.6, 0.04)	0.606 (0.607)	0.949 (0.949)	0.998 (0.998)
	(2.7, 0.04)	0.000 (0.211)	0.278 (0.455)	0.669 (0.714)
	(3.2, 0.01)	0.000 (0.324)	0.334 (0.612)	0.762 (0.854)
(25%, 25%)	(0.2, 0.01)	0.998 (0.998)	1.000 (1.000)	1.000 (1.000)
	(0.6, 0.04)	0.598 (0.600)	0.946 (0.946)	0.998 (0.998)
	(2.7, 0.04)	0.000 (0.188)	0.183 (0.425)	0.590 (0.657)
	(3.2, 0.01)	0.000 (0.307)	0.232 (0.581)	0.668 (0.807)
(25%, 50%)	(0.2, 0.01)	0.998 (0.998)	1.000 (1.000)	1.000 (1.000)
	(0.6, 0.04)	0.585 (0.590)	0.943 (0.943)	1.000 (1.000)
	(2.7, 0.04)	0.000 (0.172)	0.098 (0.387)	0.481 (0.594)
	(3.2, 0.01)	0.000 (0.266)	0.067 (0.496)	0.502 (0.710)
(50%, 50%)	(0.2, 0.01)	0.998 (0.998)	1.000 (1.000)	1.000 (1.000)
	(0.6, 0.04)	0.580 (0.585)	0.934 (0.934)	0.996 (0.996)
	(2.7, 0.04)	0.000 (0.164)	0.027 (0.339)	0.381 (0.526)
	(3.2, 0.01)	0.000 (0.242)	0.006 (0.452)	0.367 (0.634)
(75%, 50%)	(0.2, 0.01)	0.998 (0.998)	1.000 (1.000)	1.000 (1.000)
	(0.6, 0.04)	0.543 (0.548)	0.910 (0.910)	0.994 (0.994)
	(2.7, 0.04)	0.000 (0.134)	0.000 (0.260)	0.139 (0.435)
	(3.2, 0.01)	0.000 (0.190)	0.000 (0.311)	0.035 (0.474)
(75%, 75%)	(0.2, 0.01)	0.998 (0.998)	1.000 (1.000)	1.000 (1.000)
	(0.6, 0.04)	0.517 (0.841)	0.905 (0.905)	0.994 (0.994)
	(2.7, 0.04)	0.000 (0.121)	0.000 (0.223)	0.016 (0.355)
	(3.2, 0.01)	0.000 (0.160)	0.000 (0.264)	0.001 (0.409)

Due to its construction, testing non-inferiority or equivalence over an entire interval comes along with a loss of power compared to pointwise comparisons. On the other hand, the resulting statement is much stronger as in case of rejecting the null hypothesis we can conclude that the two treatments differ by less than δ throughout the whole observational period. Consequently it depends on the particular research question which procedure should be chosen.

Table 8: Simulated type I errors of the parametric non-inferiority test (15) (numbers in brackets) and the equivalence test (16) for the PH scenario (19) with $\theta_2 = (1.5, 4.9)$ at three different time points $t_0 = 1.6, 2.3, 4$ for different sample sizes and equivalence margins δ. The nominal level is chosen as $\alpha = 0.05$. Scenarios not falling under the null hypothesis are marked with "-" and numbers in bold indicate simulations on the margin of the null hypothesis.

(n_1, n_2)	$(t_0, S_2(t_0) - S_1(t_0))$	$\delta = 0.1$	$\delta = 0.15$	$\delta = 0.2$
$(20, 20)$	$(1.6, 0.1)$	**0.000 (0.049)**	-	-
	$(2.3, 0.15)$	0.000 (0.025)	**0.000 (0.051)**	-
	$(4, 0.2)$	0.000 (0.014)	0.000 (0.030)	**0.001 (0.061)**
$(50, 50)$	$(1.6, 0.1)$	**0.001 (0.049)**	-	-
	$(2.3, 0.15)$	0.000 (0.014)	**0.017 (0.045)**	-
	$(4, 0.2)$	0.000 (0.005)	0.010 (0.012)	**0.047 (0.048)**
$(100, 100)$	$(1.6, 0.1)$	**0.037 (0.037)**	-	-
	$(2.3, 0.15)$	0.000 (0.009)	**0.049 (0.041)**	-
	$(4, 0.2)$	0.000 (0.001)	0.012 (0.006)	**0.050 (0.051)**
$(150, 150)$	$(1.6, 0.1)$	**0.044 (0.054)**	-	-
	$(2.3, 0.15)$	0.001 (0.002)	**0.037 (0.040)**	-
	$(4, 0.2)$	0.000 (0.000)	0.003 (0.003)	**0.048 (0.048)**
$(250, 250)$	$(1.9, 0.1)$	**0.049 (0.049)**	-	-
	$(2.4, 0.15)$	0.003 (0.003)	**0.054 (0.054)**	-
	$(3, 0.2)$	0.000 (0.000)	0.005 (0.005)	**0.055 (0.055)**

Table 9: Simulated type I errors of the non-parametric approach for the non-inferiority test (numbers in brackets) and the equivalence test for the PH scenario (19) with $\theta_2 = (1.5, 4.9)$ at three different time points for different sample sizes and equivalence margins. The nominal level is chosen as $\alpha = 0.05$. Scenarios not falling under the null hypothesis are marked with " $-$ " and numbers in bold indicate simulations on the margin of the null hypothesis.

(n_1, n_2)	$(t_0, S_2(t_0) - S_1(t_0))$	$\delta = 0.1$	$\delta = 0.15$	$\delta = 0.2$
	$(1.6, 0.1)$	**0.000 (0.064)**	-	-
$(20, 20)$	$(2.3, 0.15)$	0.000 (0.030)	**0.000 (0.053)**	-
	$(4, 0.2)$	0.000 (0.018)	0.000 (0.030)	**0.000 (0.052)**
	$(1.6, 0.1)$	**0.000 (0.059)**	-	-
$(50, 50)$	$(2.3, 0.15)$	0.000 (0.014)	**0.000 (0.053)**	-
	$(4, 0.2)$	0.000 (0.060)	0.000 (0.018)	**0.028 (0.043)**
	$(1.6, 0.1)$	**0.012 (0.037)**	-	-
$(100, 100)$	$(2.3, 0.15)$	0.000 (0.005)	**0.047 (0.051)**	-
	$(4, 0.2)$	0.000 (0.001)	0.009 (0.014)	**0.044 (0.044)**
	$(1.6, 0.1)$	**0.052 (0.058)**	-	-
$(150, 150)$	$(2.3, 0.15)$	0.002 (0.005)	**0.030 (0.030)**	-
	$(4, 0.2)$	0.000 (0.001)	0.003 (0.003)	**0.053 (0.053)**
	$(1.6, 0.1)$	**0.035 (0.035)**	-	-
$(250, 250)$	$(2.3, 0.15)$	0.001 (0.001)	**0.044 (0.044)**	-
	$(4, 0.2)$	0.000 (0.000)	0.001 (0.001)	**0.050 (0.050)**

Table 10: Simulated type I errors of the parametric non-inferiority test (15) (numbers in brackets) and the equivalence test (16) for the NPH scenario (20) with $\theta_2 = (2, 2.5)$ at three different time points $t_0 = 1.9, 2.4, 3$ for different sample sizes and equivalence margins δ. The nominal level is chosen as $\alpha = 0.05$. Scenarios not falling under the null hypothesis are marked with "-" and numbers in bold indicate simulations on the margin of the null hypothesis.

(n_1, n_2)	$(t_0, S_1(t_0) - S_2(t_0))$	$\delta = 0.1$	$\delta = 0.15$	$\delta = 0.2$
$(20, 20)$	$(1.9, 0.1)$	**0.000 (0.060)**	-	-
	$(2.4, 0.15)$	0.000 (0.029)	**0.000 (0.049)**	-
	$(3, 0.2)$	0.000 (0.016)	0.000 (0.021)	**0.002 (0.061)**
$(50, 50)$	$(1.9, 0.1)$	**0.000 (0.053)**	-	-
	$(2.4, 0.15)$	0.000 (0.011)	**0.008 (0.052)**	-
	$(3, 0.2)$	0.000 (0.003)	0.007 (0.014)	**0.048 (0.048)**
$(100, 100)$	$(1.9, 0.1)$	**0.002 (0.057)**	-	-
	$(2.4, 0.15)$	0.000 (0.008)	**0.055 (0.055)**	-
	$(3, 0.2)$	0.000 (0.001)	0.006 (0.006)	**0.038 (0.038)**
$(150, 150)$	$(1.9, 0.1)$	**0.043 (0.053)**	-	-
	$(2.4, 0.15)$	0.004 (0.006)	**0.041 (0.041)**	-
	$(3, 0.2)$	0.000 (0.000)	0.005 (0.005)	**0.049 (0.049)**
$(250, 250)$	$(1.9, 0.1)$	**0.042 (0.042)**	-	-
	$(2.4, 0.15)$	0.000 (0.000)	**0.051 (0.051)**	-
	$(3, 0.2)$	0.000 (0.000)	0.002 (0.002)	**0.051 (0.051)**

Table 11: Simulated type I errors of the non-parametric approach for the non-inferiority test (numbers in brackets) and the equivalence test for the NPH scenario (20) with $\theta_2 = (2, 2.5)$ at three different time points for different sample sizes and equivalence margins. The nominal level is chosen as $\alpha = 0.05$. Scenarios not falling under the null hypothesis are marked with " $-$ " and numbers in bold indicate simulations on the margin of the null hypothesis.

(n_1, n_2)	$(t_0, S_1(t_0) - S_2(t_0))$	$\delta = 0.1$	$\delta = 0.15$	$\delta = 0.2$
$(20, 20)$	$(1.9, 0.1)$	**0.000 (0.057)**	-	-
	$(2.4, 0.15)$	0.000 (0.034)	**0.000 (0.057)**	-
	$(3, 0.2)$	0.000 (0.015)	0.000 (0.027)	**0.000 (0.056)**
$(50, 50)$	$(1.9, 0.1)$	**0.000 (0.056)**	-	-
	$(2.4, 0.15)$	0.000 (0.007)	**0.000 (0.048)**	-
	$(3, 0.2)$	0.000 (0.004)	0.000 (0.013)	**0.030 (0.048)**
$(100, 100)$	$(1.9, 0.1)$	**0.000 (0.051)**	-	-
	$(2.4, 0.15)$	0.000 (0.010)	**0.036 (0.043)**	-
	$(3, 0.2)$	0.000 (0.002)	0.009 (0.009)	**0.045 (0.045)**
$(150, 150)$	$(1.9, 0.1)$	**0.001 (0.048)**	-	-
	$(2.4, 0.15)$	0.000 (0.005)	**0.054 (0.054)**	-
	$(3, 0.2)$	0.000 (0.000)	0.010 (0.010)	**0.047 (0.047)**
$(250, 250)$	$(1.9, 0.1)$	**0.054 (0.058)**	-	-
	$(2.4, 0.15)$	0.003 (0.003)	**0.050 (0.050)**	-
	$(3, 0.2)$	0.000 (0.000)	0.003 (0.003)	**0.054 (0.054)**

Table 12: Simulated type I errors of the parametric equivalence test and the corresponding non-inferiority test (numbers in brackets) over the entire time range for the PH scenario (19) with $\theta_2 = (1.5, 4.9)$ and the NPH scenario (20) with $\theta_2 = (2, 2.5)$. The maximum distance is 0.2 for both scenarios and hence the last column corresponds to the situation on the margin of the null hypothesis (numbers in bold). The nominal level is chosen as $\alpha = 0.05$.

(n_1, n_2)	Scenario	$\delta = 0.1$	$\delta = 0.15$	$\delta = 0.2$
$(20, 20)$	PH	0.000 (0.004)	0.000 (0.010)	**0.000 (0.030)**
	NPH	0.000 (0.005)	0.000 (0.016)	**0.000 (0.042)**
$(50, 50)$	PH	0.000 (0.001)	0.000 (0.007)	**0.030 (0.034)**
	NPH	0.000 (0.001)	0.000 (0.008)	**0.022 (0.039)**
$(100, 100)$	PH	0.000 (0.000)	0.004 (0.004)	**0.041 (0.041)**
	NPH	0.000 (0.002)	0.002 (0.003)	**0.041 (0.043)**
$(150, 150)$	PH	0.000 (0.000)	0.003 (0.003)	**0.039 (0.039)**
	NPH	0.000 (0.000)	0.002 (0.002)	**0.040 (0.040)**

References

Com-Nougue, C., Rodary, C., and Patte, C. (1993). How to establish equivalence when data are censored: a randomized trial of treatments for b non-hodgkin lymphoma. *Statistics in medicine*, 12(14):1353–1364.

Table 13: Simulated power of the parametric equivalence test and the corresponding non-inferiority test (numbers in brackets) for the NPH scenario (20) with $\theta_2 = (2, 3.4)$, the PH scenario (19) with $\theta_2 = (1.5, 3.7)$ and identical survival curves, that is $\theta_2 = \theta_1$ (from top to bottom). The nominal level is chosen as $\alpha = 0.05$.

(n_1, n_2)	$\max_{t\in\mathcal{T}} S_1(t,\theta_1) - S_2(t,\theta_2)$	$\delta = 0.1$	$\delta = 0.15$	$\delta = 0.2$
$(20, 20)$	0.07	0.000 (0.057)	0.000 (0.121)	0.000 (0.228)
	0.05	0.000 (0.046)	0.000 (0.106)	0.000 (0.207)
	0	0.000 (0.090)	0.000 (0.183)	0.000 (0.317)
$(50, 50)$	0.07	0.000 (0.093)	0.003 (0.241)	0.190 (0.464)
	0.05	0.000 (0.088)	0.013 (0.238)	0.292 (0.446)
	0	0.000 (0.204)	0.014 (0.430)	0.352 (0.662)
$(100, 100)$	0.07	0.000 (0.130)	0.163 (0.408)	0.646 (0.748)
	0.05	0.000 (0.148)	0.343 (0.428)	0.738 (0.754)
	0	0.000 (0.397)	0.457 (0.726)	0.852 (0.929)
$(150, 150)$	0.07	0.011 (0.161)	0.369 (0.541)	0.859 (0.885)
	0.05	0.087 (0.207)	0.564 (0.585)	0.890 (0.891)
	0	0.146 (0.545)	0.761 (0.880)	0.971 (0.985)
$(200, 200)$	0.07	0.026 (0.189)	0.547 (0.656)	0.942 (0.948)
	0.05	0.201 (0.262)	0.705 (0.709)	0.955 (0.955)
	0	0.356 (0.672)	0.902 (0.948)	0.996 (0.998)
